# Bioinspired Engineering of Streamlined Skeletal Interoception: Neural Bioprinted Piezoelectric Scaffolds for Neuro‐Vascularized Bone Regeneration

**DOI:** 10.1002/advs.202524181

**Published:** 2026-02-15

**Authors:** Yingze Su, Haomin Wang, Weixi Liu, Kangming Chen, Yiyang Min, Jinbo Zhu, Xueyang Li, Anning Su, Hao Yang, Lei Yang, Yun Ji, Yuxin Zhang, Jisi Zheng, Chi Yang, Chuanglong He, Tao Li, Shuo Chen, Tao Wu, Xiaodong Chen

**Affiliations:** ^1^ Department of Orthopaedics Xinhua Hospital affiliated to Shanghai Jiao Tong University School of Medicine Shanghai People's Republic of China; ^2^ State Key Lab of Tropic Ocean Engineering Materials and Materials Evaluation Hainan University Haikou People's Republic of China; ^3^ Department of Orthopaedics Huashan Hospital Fudan University Shanghai People's Republic of China; ^4^ Department of Otorhinolaryngology Head and Neck Surgery Shanghai Ninth People's Hospital Shanghai Jiao Tong University School of Medicine Shanghai People's Republic of China; ^5^ Department of Orthopaedics the Second Hospital of Dalian Medical University Dalian People's Republic of China; ^6^ Department of Biomedical Engineering University of Shanghai for Science and Technology Shanghai People's Republic of China; ^7^ Department of Orthopaedics Shanghai Ninth People's Hospital Shanghai Jiao Tong University School of Medicine Shanghai People's Republic of China; ^8^ State Key Laboratory of Advanced Fiber Materials College of Biological Science and Medical Engineering Donghua University Shanghai People's Republic of China; ^9^ Department of Pain Medicine Shanghai Ninth People's Hospital Shanghai Jiao Tong University School of Medicine Shanghai People's Republic of China; ^10^ Department of Oral Surgery Shanghai Ninth People's Hospital Shanghai Jiao Tong University School of Medicine Shanghai People's Republic of China; ^11^ Shanghai University of Medicine & Health Sciences Shanghai People's Republic of China

**Keywords:** 3D printing, bone regeneration, calcitonin gene‐related peptide, piezoelectric biomaterials, sensory nerve

## Abstract

The skeletal interoception system that orchestrates bone homeostasis and regeneration through a coordinated “afferent‐integration‐efferent” reflex arc has been overlooked in bone tissue engineering. The dorsal root ganglion (DRG) neurons constitute the hubs of this system. Moreover, beyond their canonical role within the interoceptive pathway, these neurons exert a direct effector role by secreting calcitonin gene‐related peptide (CGRP) to promote bone repair. However, current sensory nerve‐targeted strategies in bone tissue engineering remain static, bypassing the dynamic reflex arc and failing to establish an autonomous functional system. To address this, a streamlined interoception unit was engineered using neural bioprinting of DRG neurons within piezoelectric scaffolds. Upon sensing ultrasound (US) stimulation, the piezoelectric poly(l‐lactide) (PLLA) component mediates mechanoelectrical coupling, triggering a Ca^2^^+^ influx‐induced effector response in the incorporated DRG neurons that enhances CGRP secretion and expression. The secreted CGRP subsequently promotes osteogenesis and angiogenesis in vitro and accelerates neuro‐vascularized bone regeneration in a rat femoral condyle defect model. This study established a bioinspired platform with a self‐contained “sensor‐effector” circuit, offering a novel engineered strategy for bone regeneration.

## Graphical abstract

This study develops neural bioprinted piezoelectric scaffolds which construct local “sensor‐effector” circuit, inspired by the skeletal interoception system. Upon US stimulation sensing and mechanoelectrical coupling by PLLA nanofibers, the incorporated DRG neurons effect through Ca^2^^+^ influx‐induced enhanced secretion and expression of CGRP. This process significantly promotes osteogenesis and angiogenesis, offering a new strategy for neuro‐vascularized bone regeneration.

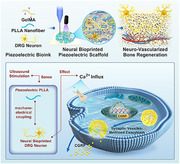

## 1 Introduction

Bone defects impose a heavy burden on society, with over two million bone‐grafting operations performed annually worldwide [1, 2]. Current clinical strategies are limited by donor availability and poor bone integration [3, 4]. Bone tissue engineering, which combines biomaterials, cells, and biofunctional molecules, is a promising alternative [5, 6]. However, their therapeutic efficacy critically depends on the adoption of strategies that faithfully replicate native biological principles governing the natural regenerative processes of bones [7, 8]. Physiologically, homeostasis and regeneration of bones are governed by a sophisticated skeletal interoception system that operates through a classic long‐loop “afferent‐integration‐efferent” reflex arc [9, 10]. Upon detection by dorsal root ganglion (DRG) neuron terminals, stimuli such as mechanical forces are transduced into electrical cues and transmitted as afferent signals to the central nervous system (CNS), which subsequently issues efferent commands, such as regulation of the autonomic nervous system, to regulate bone homeostasis [11, 12]. Crucially, DRG neurons reside in the heart of this loop. However, beyond their role as sensors for signal transmission in the skeletal interoceptive system, compelling evidence has revealed that DRG neurons can also act as potent local effectors during bone regeneration [13, 14]. They execute this effector function primarily by releasing neuropeptides, among which calcitonin gene‐related peptide (CGRP) has emerged as a key mediator that directly stimulates bone repair [15, 16]. CGRP is a 37‐amino‐acid bioactive neuropeptide without a quaternary structure and is therefore rapidly degraded in the plasma, resulting in a short half‐life [17]. Once depolarized, CGRP is released at the terminals of DRG neurons to promote osteogenesis and angiogenesis during bone regeneration [18, 19]. Despite its well‐established physiological role, the therapeutic potential of skeletal interoception has not yet been translated into mainstream bone tissue engineering paradigms. Earlier studies have mainly focused on osteo‐lineage cells, such as bone marrow‐derived mesenchymal stromal cells (BMSCs), to promote bone regeneration. However, bone defects often involve concurrent sensory nerve damage [20], which disrupts the skeletal interoception system and consequently impairs bone repair. Recent studies have begun to emphasize the role of sensory nerves in bone regeneration, leading to strategies such as CGRP‐loaded biomaterials [21, 22], gene therapy targeting CGRP receptors [23, 24], and biomaterials that regulate CGRP expression and secretion [25, 26]. However, these strategies are inherently static and passive, merely targeting final output signal (CGRP) of sensory nerves as effectors, while completely bypassing the dynamic “afferent‐integration‐efferent” reflex arc. Consequently, they do not constitute autonomous functional systems. Moreover, other drawbacks include short half‐life of CGRP [27] and the potential adverse effects of gene therapy [28]. Consequently, there is an urgent need for an intelligent strategy that can construct engineered skeletal interoception circuits that respond actively to dynamic environmental cues.

To overcome this problem, we propose a bioinspired engineering strategy centered on streamlined skeletal interoception, implemented via piezoelectric biomaterials and neural bioprinting technology. This concept functionalizes the classical interoception system by capturing its core “afferent‐integration‐efferent” logic to create a local, autonomous “sensor‐effector” circuit, thereby bypassing the need for central neural integration to enable direct, on‐demand control over regeneration at the defect site. Piezoelectric biomaterials possess the inherent functionality of mechanoelectrical coupling, responding to mechanical stimuli such as ultrasound (US) by generating electrical energy [29, 30, 31, 32], which is ideally suited to support neuronal function, as nerves naturally depolarize in response to electrical cues to execute their physiological functions [33, 34]. This inherent property enables them to function as artificial mechanosensors within this circuit by perceiving external mechanical cues. Furthermore, through mechanoelectrical coupling, these materials transform the perceived mechanical signals into localized electrical outputs, thereby fulfilling the integration function locally. Having established the ‘afferent’ and ‘integration’ modules, the next pivotal component is a living, responsive ‘effector’. This occurs when the neural bioprinting converges with the piezoelectric scaffolds to assemble the bioinspired circuit. Neural bioprinting provides a viable technological pathway for implementing this concept [35, 36]. Notably, Roversi et al. successfully bioprinted DRG neurons with high viability and minimal functional loss [37], demonstrating the feasibility of directly incorporating neural components into engineered constructs. In our design, the bioprinted DRG neurons acted as effector components, analogous to the efferent pathway of native skeletal interoception. They respond to piezoelectricity‐triggered electrical cues by releasing CGRP, which promotes osteogenesis and vascularization. This elegant design materializes the streamlined skeletal interoception concept, establishing a local, autonomous “sensor‐effector” circuit that executes the entire sequence from mechanical sensing to repair initiation directly at the injury site.

**FIGURE 1:**
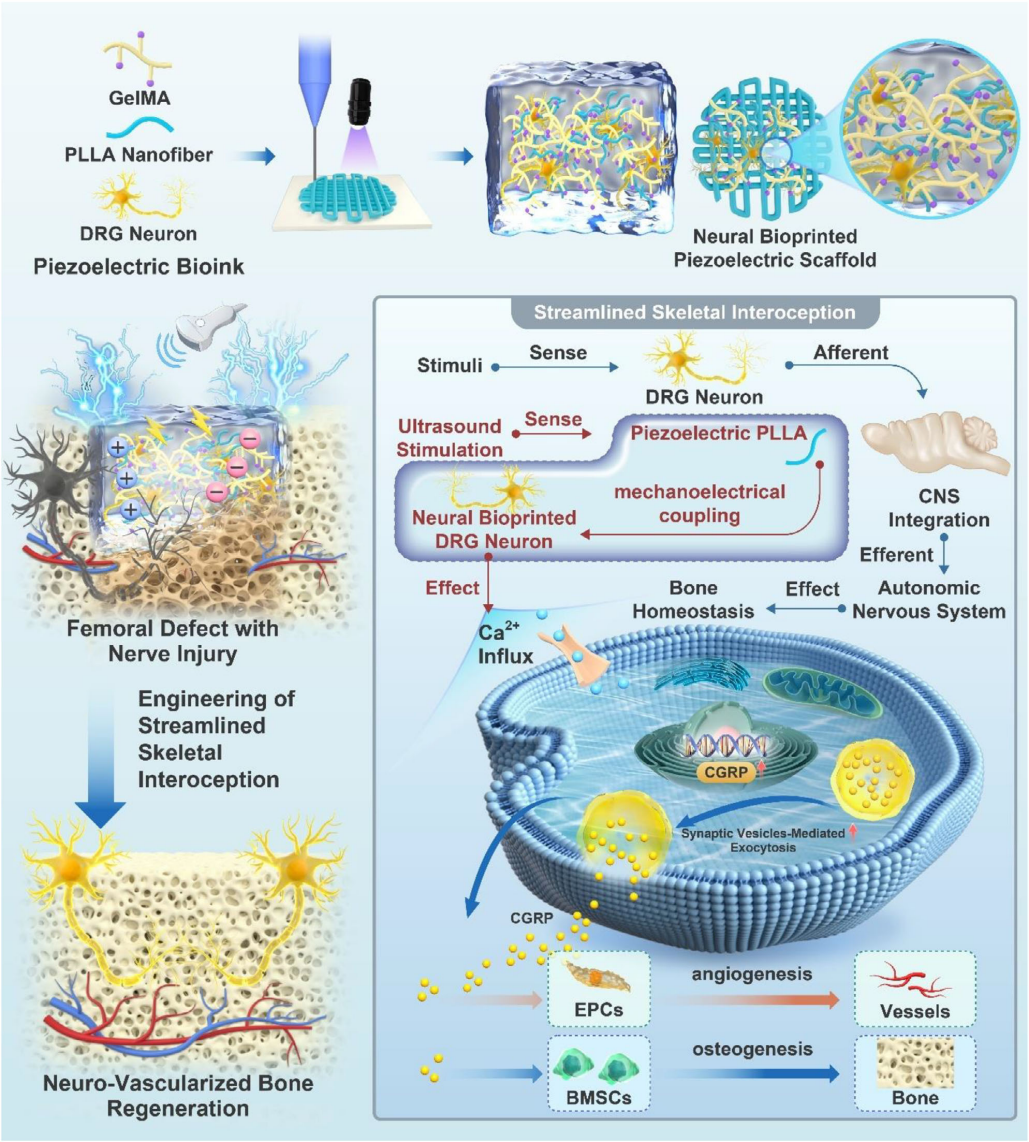
Schematic illustration of the neural bioprinted piezoelectric scaffolds promoting neuro‐vascularized bone regeneration through engineering of streamlined skeletal interoception.

In this study, we fabricated neural bioprinted piezoelectric scaffolds incorporating piezoelectric poly(l‐lactide) (PLLA) nanofibers and DRG neurons. We systematically characterized this construct and confirmed its effective sensing and mechanoelectrical coupling properties. We then demonstrated that US‐driven piezoelectric stimulation effectively promoted CGRP secretion and expression in DRG neurons, validating the effector capacity within the circuit and further elucidating the underlying mechanisms involved in calcium signaling. Subsequent in vitro assays confirmed that the enhanced osteogenic and angiogenic differentiation was mediated by CGRP release. Finally, in a rat femoral condyle defect model, the scaffold significantly promoted neuro‐vascularized bone regeneration. The overall experimental workflow is illustrated in Figure 1. This study established a functional system based on bioinspired engineering of streamlined skeletal interoception to promote neuro‐vascularized bone regeneration.

## 2 Results and Discussion

### 2.1 Preparation and Characterization of the PLLA Nanofibers

**FIGURE 2:**
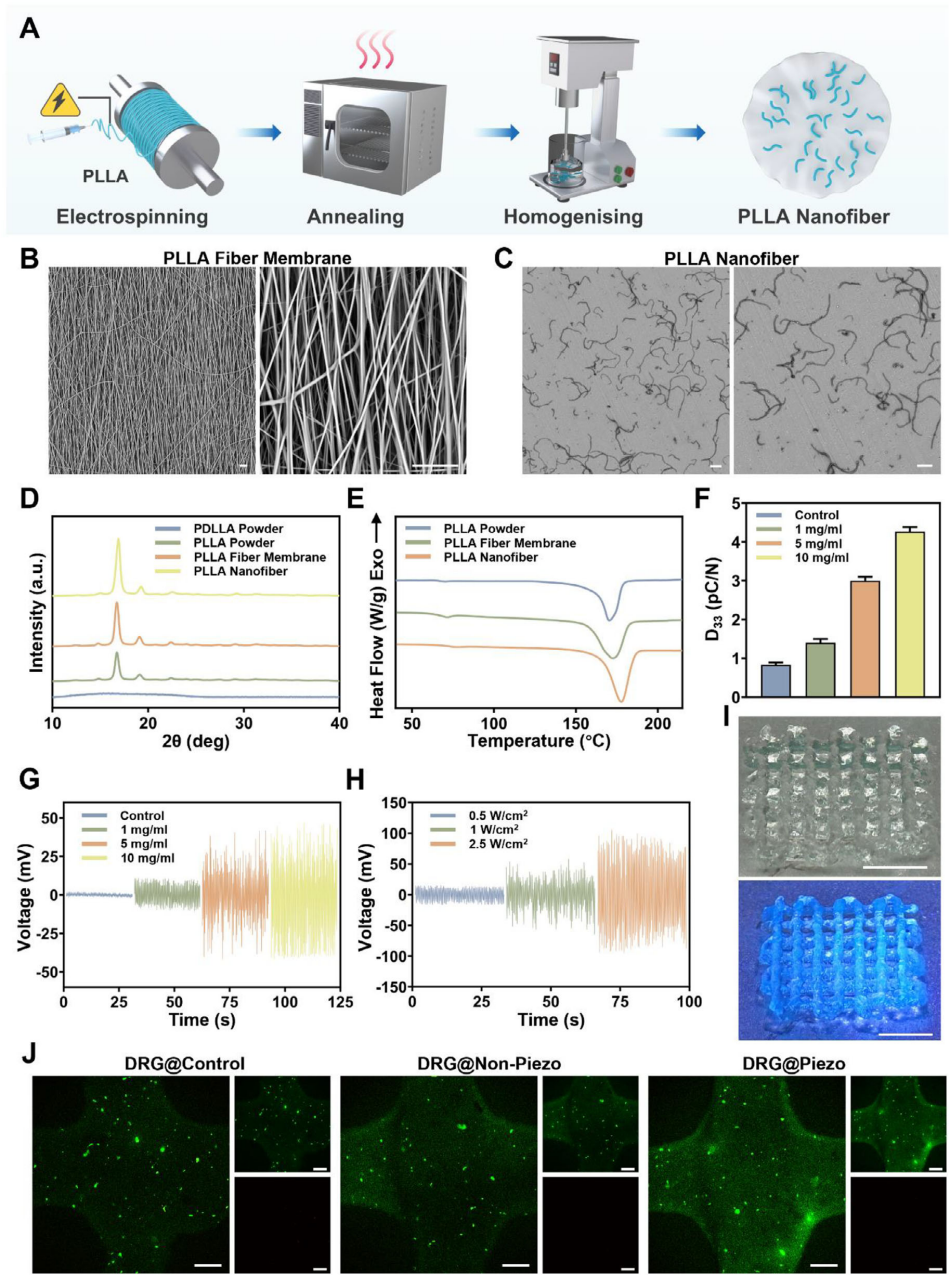
Preparation and characterization of the PLLA nanofibers and the neural bioprinted piezoelectric scaffolds. (A) Schematic of the preparation of piezoelectric PLLA nanofibers. (B) SEM images of the top view of the PLLA fiber membrane. (C) SEM images of the PLLA nanofibers. (D) XRD patterns of PDLLA powder, PLLA powder, PLLA fiber membrane, and PLLA nanofibers. (E) DSC results of PLLA powder, PLLA fiber membrane and PLLA nanofibers. (F) D_33_ coefficient of PLLA nanofiber solutions with varying concentrations (1, 5, and 10 mg ml^−1^). (G) Voltage output of the piezoelectric scaffolds with different PLLA concentrations (1, 5, and 10 mg ml^−1^) under uniform US stimulation. (H) Voltage output of the piezoelectric scaffolds with uniform concentration under different US intensities (0.25, 1, and 2.5 W cm^−2^). (I) Representative macrographs of the neural bioprinted piezoelectric scaffolds. (J) Live/dead cell staining of the 3D pioprinted DRG@Control, DRG@Non‐Piezo, and DRG@Piezo scaffolds cultured for 3 days. Viable cells are stained green (Calcein‐AM), and dead cells are stained red (Propidium Iodide). Scale bar, 20 µm(B, C), 5 mm(I), 200 µm (J).

PLLA possesses excellent biocompatibility with intrinsic piezoelectric properties while exhibiting relatively low piezoelectric activity owing to its relatively disordered molecular chain alignment [38, 39]. Previous studies have found that fibrillation and annealing processes could significantly improve piezoelectricity of PLLA through improved β‐form crystal structure and the crystallinity [40]. Therefore, in this study, we acquired PLLA nanofibers by electrospinning, annealing, and further homogenization, as shown in Figure 2A, and tested their application in the piezoelectric stimulation of neural bioprinted scaffolds.

The morphological characteristics of the PLLA electrospun fiber membranes and their nanofibers were systematically investigated using scanning electron microscopy (SEM). Figure 2B displays a top view of the oriented electrospun PLLA fiber membrane, showing uniform single‐fiber diameters of approximately 1.12 µm with consistent parallel alignment, minimal entanglement, and a macroscopic orientation degree >90%. This ordered architecture facilitates the directional alignment of the piezoelectric dipoles, thereby enhancing the piezoelectric response characteristics of the material. Following annealing and homogenized cutting processes, the membrane was segmented into discrete nanofibers. SEM results indicate the PLLA nanofiber lengths concentrated in the 60–100 µm range with no thermal damage, confirming that low‐temperature annealing avoids fiber melting or deformation. High‐magnification images further verified the smooth fiber surfaces, consistent diameters, and excellent dispersion homogeneity without agglomeration (Figure 2C). This high‐aspect‐ratio, monodisperse nanofiber structure preserves the piezoelectric β‐crystalline phase of the original fibers.

The crystallinity and thermal behavior were systematically characterized via x‐ray diffraction (XRD) and Differential Scanning Calorimetry (DSC) analyses. XRD shows that the PLLA powder exhibits characteristic diffraction peaks at 2θ ≈ 16.7° and 19.1°, indicating its crystallinity and β‐form crystal structure, while powder of poly‐ (D, L‐lactic acid) (PDLLA), which is often used as control of PLLA without piezoelectricity, exhibits only a broad diffuse peak around 2θ ≈ 16°, confirming its amorphous nature without piezoelectric ability. Compared to the original powder, the XRD of the acquired PLLA fiber membranes and nanofibers after electrospinning, annealing, and homogenizing showed an incrementally enhanced peak intensity and reduced full width at half maximum, providing clear evidence that the fibrillation and annealing processes effectively improved the crystallinity and crystal perfection of the material (Figure 2D). Further DSC exhibits a similar melting endothermic peak at 175°C for PLLA powder, fiber membrane and nanofibers, indicating that the intrinsic thermodynamic properties of the material remained unaffected by the aforementioned treatments (Figure 2E). To test the piezoelectric property of the PLLA nanofiber, we measured the D_33_ coefficient of PLLA at varying concentrations. As seen in Figure 2F, as the loading of PLLA nanofibers increased from 1 to 10 mg mL^−1^, the D_33_ value rose from 1.4 pC N^−1^ to approximately 4.3 pC N^−1^. This indicates that the high orientation of the PLLA nanofibers and the increased proportion of β‐form crystals significantly enhance the piezoelectric effect.

Collectively, these results confirmed that electrospinning combined with annealing yielded well‐crystallized piezoelectric PLLA fibers. The homogenized cutting process enables efficient PLLA nanofiber production without compromising their intrinsic properties. This synergistic structure‐process optimization strategy enabled the fabrication of piezoelectric scaffolds with integrated functionality for US sensing and mechanoelectrical coupling.

### 2.2 Preparation and Characterization of the Neural Bioprinted Piezoelectric Scaffolds

Following our demonstration of piezoelectricity in PLLA nanofibers, we prepared piezoelectric scaffolds incorporating PLLA nanofibers using 3D printing technology and evaluated their piezoelectric performance, which we further employed during in vitro and in vivo studies. As two critical determinants of piezoelectric performance, we systematically investigated the effects of nanofiber loading and US power on the piezoelectric output of the piezoelectric scaffolds. To evaluate the effect of fiber loading, four experimental groups with PLLA nanofiber concentrations of 0, 1, 5, and 10 mg mL^−1^ were established. Voltage output was measured for each group. As shown in Figure 2G, the piezoelectric output exhibited a marked increase when fiber loading rose from 0 to 1 mg mL^−1^. Further elevation to 5 mg mL^−1^ yielded continued significant enhancement. However, increasing the concentration from 5 to 10 mg mL^−1^ resulted in only a marginal improvement, with no obvious statistical difference between the two groups (Figure 2G). This demonstrates that while a higher fiber loading enhances the piezoelectric performance within a certain range, the enhancement plateaus beyond 5 mg mL^−1^. Subsequently, we examined the effects of US power (0.25, 1, and 2.5 W cm^−2^) on the selected groups. Figure 2H shows that all the output voltages increased progressively with increasing US power. Notably, output increased substantially when power escalated from 0.25 to 1 W cm^−2^, but only modestly from 1 to 2.5 W cm^−2^ (Figure 2H). Considering practical printability requirements and potential cellular damage from excessive US exposure, we selected 5 mg mL^−1^ fiber loading and 1 W cm^−2^ US power as optimal parameters for subsequent biological experiments. In addition, US‐responsive voltage output testing of the piezoelectric scaffolds was conducted using cyclic on/off US triggering (Figure S1). When the US was cyclically toggled, the scaffolds exhibited immediate voltage generation upon activation with a stable signal amplitude, followed by rapid signal decay upon cessation. This rapid switching behavior confirms excellent US responsiveness, demonstrating strong viability for practical applications requiring on‐demand mechanoelectrical transduction. Moreover, for subsequent in vitro and in vivo studies, non‐Piezo scaffolds incorporating PDLLA nanofibers into gelatin methacryloyl (GelMA) and control scaffolds consisting of pure GelMA were also 3D printed. The details of the scaffolds used in this study are listed in Table 1.

**TABLE 1:** Abbreviation Details Table for Scaffolds Involved.

	GelMA	PDLLA	PLLA	DRG neurons
Control	+	—	—	—
Non‐Piezo	+	+	—	—
Piezo	+	—	+	—
DRG@Control	+	—	—	+
DRG@Non‐Piezo	+	+	—	+
DRG@Piezo	+	—	+	+

After demonstrating the piezoelectricity of the PLLA nanofiber‐incorporated scaffolds, we further explored the feasibility of preparing DRG neurons‐incorporated scaffolds using 3D bioprinting technology and characterized the construct. GelMA, synthesized by the reaction of gelatin with methacrylic anhydride, possesses excellent sensitivity to temperature, printability, and biocompatibility. Therefore, it is widely used as a substrate for bioinks in 3D bioprinting [41]. Therefore, GelMA‐based bioinks were used in this study. Representative macrographs of the neural bioprinted scaffolds are shown in Figure 2I. For biocompatibility testing, live‐dead staining of the bioprinted scaffolds was conducted after in vitro culture for 3 days. DRG@Control, DRG@Non‐Piezo, and DRG@Piezo scaffolds exhibited excellent cell viability with few dead cells, suggesting high viability of DRG neurons bioprinted with GelMA‐based bioinks and less cytotoxicity of PLLA or PDLLA nanofibers (Figure 2J). CGRP is a critical mediator of its effector role as a representative neuropeptide in DRG neurons. To test the functional activity of the different scaffolds, we performed enzyme‐linked immunosorbent assay (ELISA) to detect the secretion of CGRP from the different neural bioprinted scaffolds at different time points. No obvious difference in CGRP concentration within the supernatant was observed among these groups, whereas CGRP levels in all three groups exhibited an increasing trend by day 7, possibly due to extended neural axons, followed by a slight decrease by day 14, and remained relatively constant at the later time points of day 21 and day 28 (Figure S2).

This result reveals the piezoelectric properties of PLLA nanofibers when incorporated into scaffolds, and appropriate parameters regarding fiber loading and US power were determined by considering the biological effect, practical printability, and potential cellular damage. Additionally, the neural bioprinting of DRG neurons with a GelMA‐based bioink exhibited excellent cytocompatibility and biological functionality, regardless of whether they were incorporated with PDLLA or PLLA nanofibers. These findings confirm that the PLLA nanofibers within the piezoelectric scaffolds can sense US stimulation and convert it into electrical signals via mechanoelectrical coupling, which is a critical step toward realizing sensor function in the proposed interoception circuit. Moreover, the fabrication of neural bioprinted piezoelectric scaffolds established the basis for our subsequent investigation into the effector functions of the incorporated DRG neurons, which is critical for engineering a functional streamlined skeletal interoception unit.

### 2.3 Piezoelectric Stimulation Enhances the Secretion and Expression of CGRP Through Enhanced Influx of Ca^2+^

Studies have shown that electrical stimulation can promote the secretion and expression of CGRP in DRG neurons through Ca^2+^‐related signaling pathways [26, 42]. As a neuropeptide, synaptic vesicle‐mediated exocytosis is the primary mechanism underlying the release [43]. Therefore, we tested whether the electrical cues generated from the piezoelectric PLLA nanofibers under US stimulation enhanced synaptic vesicle‐mediated exocytosis and expression of CGRP by promoting Ca^2^^+^ influx into DRG neurons.

**FIGURE 3:**
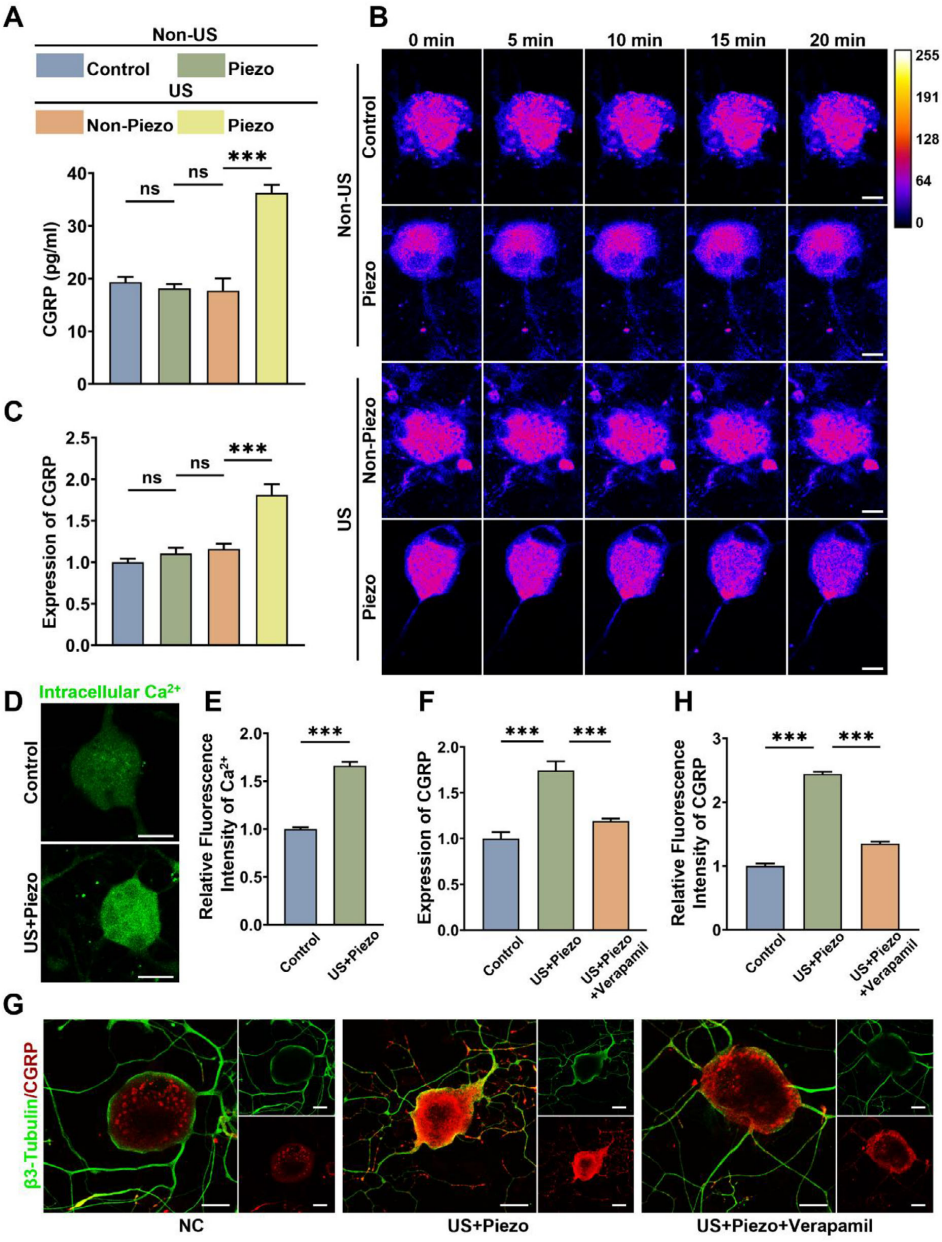
The effects of piezoelectric stimulation on secretion and expression of CGRP in DRG neurons. (A) ELISA for CGRP concentration of different neural bioprinted scaffolds with or without US stimulation (n = 3, mean ± s.d.). (B) Images of synaptic vesicle exocytosis in DRG neurons cultured beneath different scaffolds with or without US stimulation, false color images were generated using ImageJ. (C) Relative mRNA expression levels of CGRP in DRG neurons cultured beneath different scaffolds with or without US stimulation (n = 3, mean ± s.d.). (D) Intracellular calcium IF of DRG neurons with or without piezoelectric stimulation. (E) Semi‐quantitative analysis of intracellular calcium MFI (n = 3, mean ± s.d.). (F) Relative mRNA expression levels of CGRP in DRG neurons from different groups: Control, US+Piezo, US+Piezo+Verapamil (n = 3, mean ± s.d.). (G) IF staining showing β3‐Tubulin (green) and CGRP (red) in DRG neurons from different groups (n = 3, mean ± s.d.). (H) Relative fluorescence intensity of CGRP (n = 3, mean ± s.d.). n = number of biologically independent samples. Scale bar, 10 µm(B), 50 µm (D, G). (ns, not significant; ^***^ p<0.0001).

To test the effect of piezoelectric stimulation on CGRP secretion from DRG neurons incorporated in scaffolds, we stimulated the DRG@Piezo and DRG@Non‐Piezo scaffolds with an US generator and compared them with the DRG@Control and DRG@Piezo scaffolds without US stimulation. Figure 3A shows the concentration of CGRP in the supernatants from the above groups using ELISA. Only the US + Piezo group, which symbolizes piezoelectric stimulation, significantly increased the concentration of CGRP released from DRG neurons incorporated in the scaffolds, whereas PLLA or US stimulation alone exerted no effect. To more clearly observe the synaptic vesicles‐mediated exocytosis process and intracellular Ca^2+^, we cultured DRG neurons in Petri dishes and placed different groups of scaffolds on top of the adherent cells, combined with non‐US or US stimulation, in subsequent experiments. Similarly, only piezoelectric stimulation combining PLLA and US stimulation stimulated the secretion of CGRP from Petri dish‐cultured DRG neurons (Figure S3). Furthermore, we visualized the synaptic vesicles in DRG neurons using the fluorescent dye FM1‐43. In groups without piezoelectric stimulation, the fluorescence intensity of synaptic vesicles in DRG neurons remained relatively constant during the observation period. In contrast, with piezoelectric stimulation, the fluorescence intensity markedly decreased as the observation period progressed, indicating enhanced synaptic vesicle‐mediated exocytosis of CGRP (Figure 3B). However, as a short half‐life neuropeptide without sufficient reserves, simply promoting the secretion of CGRP cannot ensure its persistent effect. Therefore, we examined the effect on CGRP expression by piezoelectric stimulation through quantitative polymerase chain reaction (qPCR). As shown in Figure 3C, piezoelectric stimulation upregulated CGRP mRNA expression in the DRG neurons. Additionally, we performed real‐time membrane potential measurements on DRG neurons to characterize the effect of piezoelectric stimulation on their depolarization. The results showed that piezoelectric stimulation induced by US could evoke depolarization of DRG neurons (Figure S4).

As a neuropeptide, CGRP expression and secretion are closely linked to intracellular Ca^2^^+^ levels [44]. To explore intracellular Ca^2+^ concentration, we conducted calcium imaging and semi‐quantitative analysis of DRG neurons with or without piezoelectric stimulation. Figure 3D,E show that piezoelectric stimulation induced a remarkable increase in Ca^2+^ in the DRG neurons. Aiming at investigating the role of Ca^2+^ in promoting the secretion and expression of CGRP, verapamil was applied as blocker of L‐type calcium channels. ELISA for CGRP showed that piezoelectric stimulation could notably enhance the CGRP concentration of the supernatant derived from both scaffolds‐incorporated and petri dish‐cultured DRG neurons, while blocking the calcium influx using verapamil significantly reduced the enhancement (Figure S5A, B). Additionally, qPCR and immunofluorescence (IF) assays to detect CGRP expression at the mRNA and protein levels showed similar trends (Figure 3F–H). Therefore, Ca^2+^ influx serves as a mediator of enhanced CGRP secretion and expression following piezoelectric stimulation.

This result reveals that within the neural bioprinted piezoelectric scaffolds, the incorporated DRG neurons effectively functioned as effector components. Upon receiving US‐evoked piezoelectric signals, these neurons mounted a robust Ca^2^^+^‐dependent response as an effector, characterized by significant enhancement in both the secretion and expression of CGRP. Critically, the observed upregulation of CGRP at the mRNA level ensures the sustained synthesis of this neuropeptide, overcoming its intrinsically short half‐life and guaranteeing a long‐lasting biological effect. Moreover, this result successfully demonstrates the feasibility of the neural bioprinted piezoelectric scaffolds in establishing a local “sensor‐effector” circuit as an engineered streamlined skeletal interoception unit. This functional validation provides a pivotal foundation for subsequent investigations of neuro‐vascularized bone regeneration mediated by this bioinspired platform.

### 2.4 Evaluation of the In Vitro Osteogenic Potentials of the Neural Bioprinted Piezoelectric Scaffolds

BMSCs play an essential role in bone homeostasis and regeneration through osteogenic differentiation, which can directly promote bone defect repair [45]. Recent studies have found that the sensory nerve and its neuropeptide CGRP play important regulatory roles in the osteogenic differentiation of BMSCs, whereas previous studies on bone tissue engineering mainly focused on the direct regulation of BMSCs [23, 46]. Herein, using conditioned medium, we investigated the promotional effect of DRG@Control and US‐stimulated DRG@Piezo scaffolds on the osteogenic differentiation of BMSCs.

**FIGURE 4:**
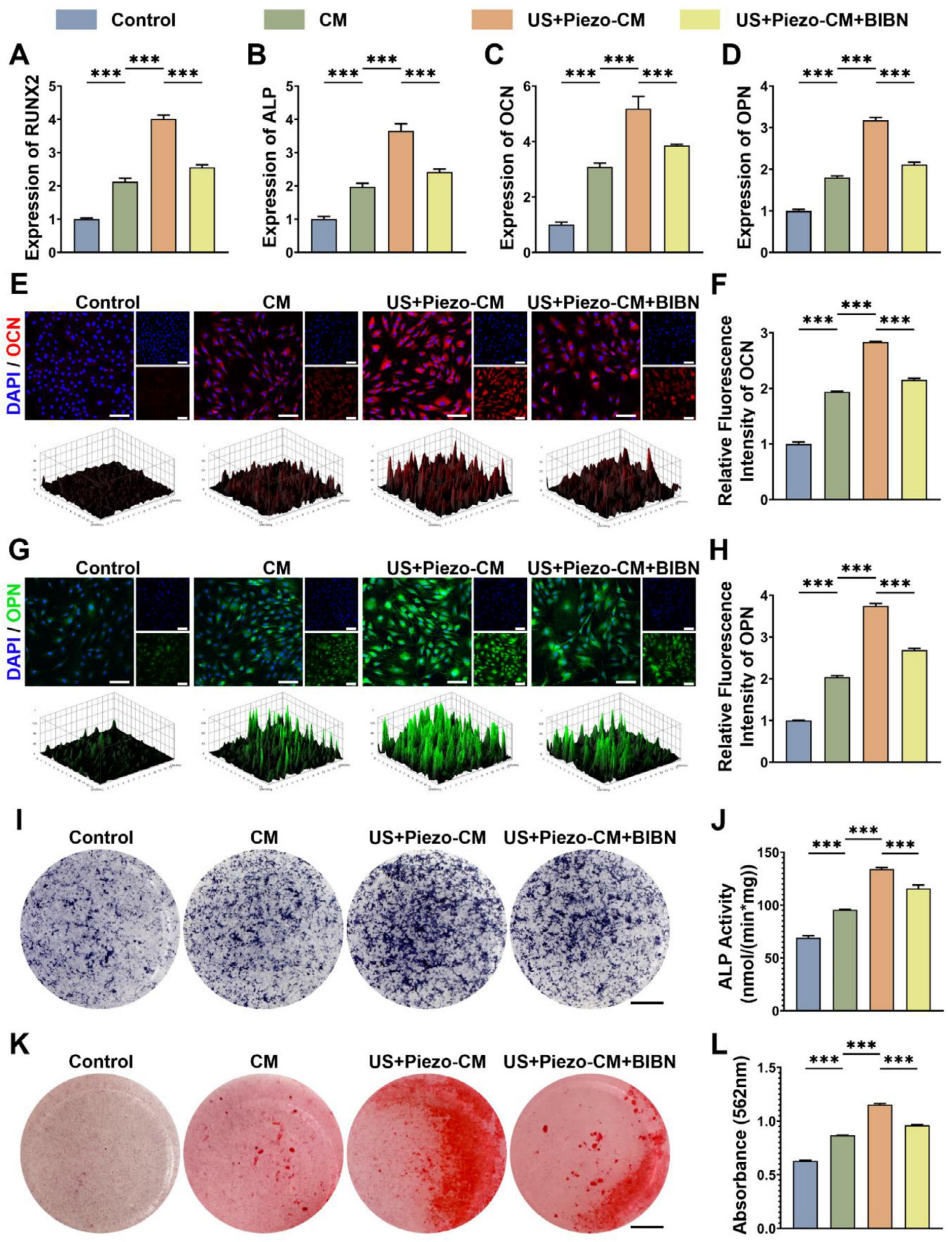
The effects of the neural bioprinted piezoelectric scaffolds on osteogenic differentiation of BMSCs. (A–D) Relative mRNA expression levels of osteogenic markers (RUNX2, ALP, OCN, OPN) in BMSCs from different groups: Control, CM, US+Piezo‐CM, US+Piezo‐CM+BIBN4096 (n = 3, mean ± s.d.). (E) IF micrographs depicting OCN (red) in BMSCs from different groups, counterstained with DAPI (blue) for nuclei. (F) Relative fluorescence intensity of OCN (n = 3, mean ± s.d.). (G) IF micrographs depicting OPN (green) in BMSCs from different groups, counterstained with DAPI (blue) for nuclei. (H) Relative fluorescence intensity of OPN (n = 3, mean ± s.d.). (I) ALP staining of BMSCs in different groups on day 14 of coculture (J) ALP activity testing of BMSCs in different groups (n = 3, mean ± s.d.). (K) ARS staining of BMSCs in different groups on day 21 of coculture. (L) Quantitative analysis of OD values at 562 nm of mineralized matrix of different groups (n = 3, mean ± s.d.). n = number of biologically independent samples. Scale bar, 100 µm (E, G), 5 mm (I, K). ^(***^ p<0.0001).

First, the CCK‐8 assay was performed to examine the effects of different conditioned medium on BMSCs viability. The results indicated no significant difference in BMSCs viability among different groups at 1, 3, and 5 days (Figure S6). To test the expression of osteogenic markers (RUNX2, ALP, OCN, and OPN) at the mRNA level, we conducted qPCR assays after coculture with the control or conditioned medium for 3 days (Figure 4A–D). Upon promotion of the mRNA expression of the above osteogenic markers by conditioned medium extracted from DRG@Control scaffolds, conditioned medium extracted from DRG@Piezo scaffolds under US stimulation further enhanced the promoting effect. To examine the role of CGRP in this phenomenon, inhibitory experiments were performed using BIBN4096, a CGRP antagonist. As shown in Figure 4A–D, the promoting effects were rescued by BIBN4096, indicating the central role of CGRP in promoting osteogenic differentiation. Moreover, to verify osteogenic differentiation at the protein level, we performed IF of OCN and OPN and semi‐quantitative analysis after coculture for 7 days (Figure 4E–H). A similar trend in the relative fluorescence intensities of OCN and OPN was observed compared to that of the qPCR assay.

The assessment of ALP, a key marker of early osteogenic differentiation and of matrix mineralization, a definitive late‐stage marker, served to monitor osteogenic progression. ALP staining, activity, and Alizarin Red S (ARS) staining were evaluated at 14 and 21 days post‐coculture. ALP expression and activity were enhanced in the coculture with conditioned medium extracted from DRG@Control scaffolds, whereas coculture with conditioned medium extracted from DRG@Piezo scaffolds under US stimulation further enhanced ALP expression and activity (Figure 4I,J). ARS staining and relative quantitative analysis of the mineralized matrix showed similar patterns (Figure 4K,L). Both the enhancement of ALP activity and mineralized matrix were impaired by the antagonist of CGRP, BIBN4096, validating the central role of CGRP.

Based on the presented results, we demonstrated that the neural bioprinted scaffolds alone could promote the osteogenic differentiation of BMSCs through the effector role of the DRG neurons incorporated by CGRP. However, upon US stimulation, neural bioprinted piezoelectric scaffolds may further enhance osteogenesis. This confirmed the extraordinary capability of the engineered skeletal interoception unit to promote osteogenesis by establishing a local “sensor‐effector” circuit.

### 2.5 Evaluation of the In Vitro Angiogenic Properties of the Neural Bioprinted Piezoelectric Scaffolds

Reconstruction of the vascular network provides nutrients, growth factors, and a path for recruitment of BMSCs. Previous studies have shown that the sensory nerve, along with the neuropeptide CGRP, positively affects angiogenesis. In this part of the study, we investigated the proangiogenic effect of our engineered streamlined skeletal interoception strategy using a conditioned medium to examine the promotive effect of DRG@Control and US‐stimulated DRG@Piezo scaffolds on endothelial progenitor cells (EPCs) angiogenesis mediated by the incorporated DRG neurons acting as effectors. Furthermore, we characterized the involvement of CGRP in this process.

**FIGURE 5:**
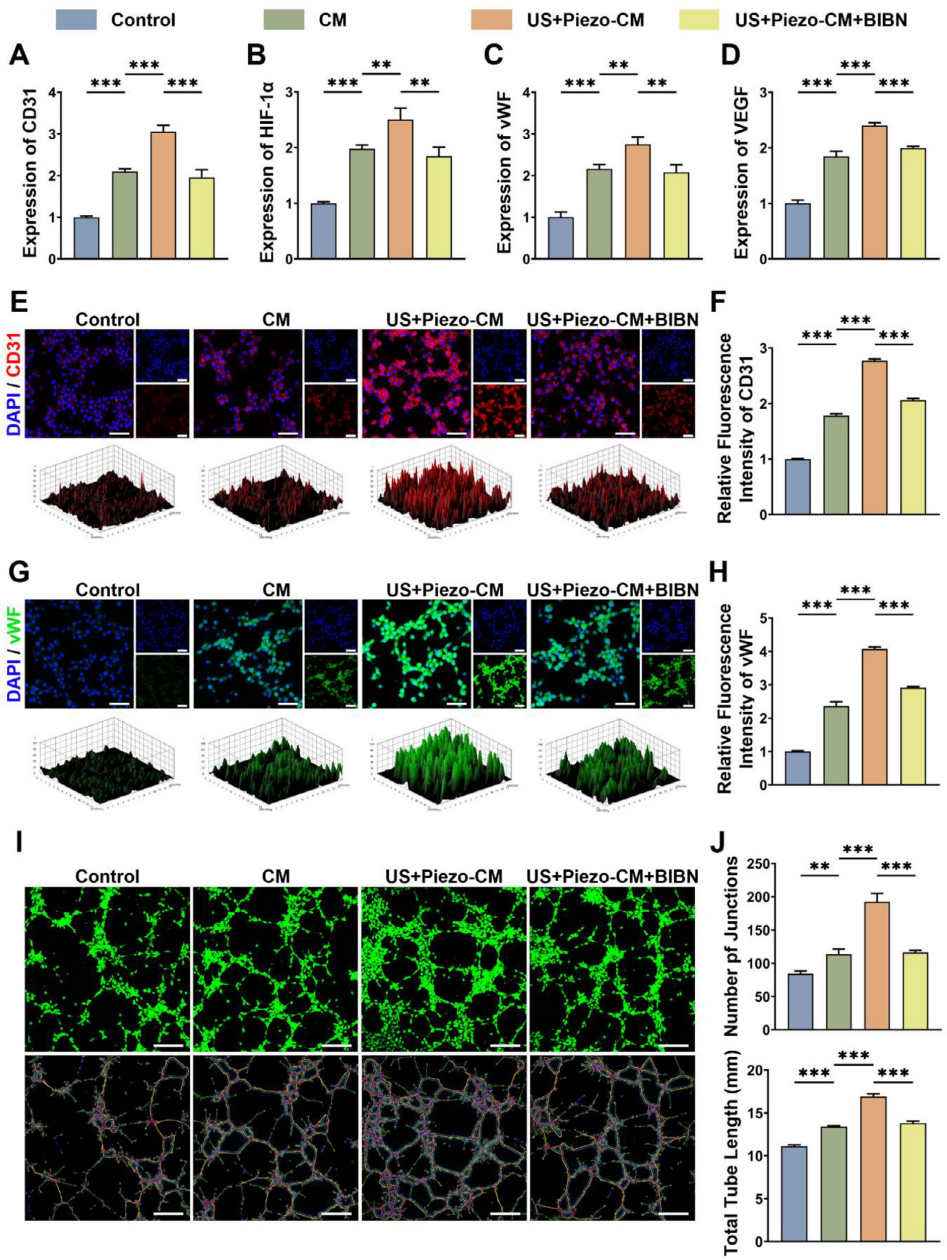
The effects of neural bioprinted piezoelectric scaffolds on angiogenesis of EPCs. (A–D) Relative mRNA expression levels of angiogenic markers (CD31, Hif‐1α, vWF, VEGF) in EPCs from different groups: Control, CM, US+Piezo‐CM, US+Piezo‐CM+BIBN4096 (n = 3, mean ± s.d.). (E) IF micrographs depicting CD31 (red) in EPCs from different groups, counterstained with DAPI (blue) for nuclei. (F) Relative fluorescence intensity of CD31 (n = 3, mean ± s.d.). (G) IF micrographs depicting vWF (green) in EPCs from different groups, counterstained with DAPI (blue) for nuclei. (H) Relative fluorescence intensity of vWF (n = 3, mean ± s.d.). (I) Tube formation assay of EPCs stained by Calcein‐AM. (J) Quantitative analysis regarding number of junctions and total tube length (n = 3, mean ± s.d.). n = number of biologically independent samples. Scale bar, 50 µm (E, G), 200 µm (I). (^**^ p<0.001; ^***^ p<0.0001).

First, the effect of different conditioned medium on EPCs viability was first assessed via CCK‐8 assay. The results revealed no significant differences in viability among the groups following 1, 3, or 5 days of exposure (Figure S7). To test the angiogenic markers (CD31, Hif‐1α, vWF, VEGF) expression at the mRNA level, we conducted qPCR assays after coculture with the control or conditioned medium for 3 days (Figure 5A–D). Upon promotion of the mRNA expression of the above angiogenic markers by conditioned medium extracted from DRG@Control scaffolds, conditioned medium extracted from DRG@Piezo scaffolds under US stimulation further enhanced the promoting effect. To verify the role of CGRP in this phenomenon, inhibitory experiments using BIBN4096 were performed. As shown in Figure 5A–D, the promotional effects were rescued by BIBN4096, indicating the central role of CGRP in promoting angiogenic differentiation. Moreover, to verify angiogenic differentiation at the protein level, we performed IF of CD31 and vWF and semi‐quantitative analysis after coculture for 3 days (Figure 5E–H). Similar trends in the relative fluorescence intensity of CD31 and vWF were observed when compared to the qPCR assay.

In addition to the detection of angiogenic marker expression, migration and tube formation ability also symbolize angiogenic properties, which can be assessed using transwell and tube formation assays. The number of migrated cells increased with chemotaxis in the conditioned medium extracted from DRG@Control scaffolds, whereas the conditioned medium extracted from DRG@Piezo scaffolds under US stimulation further enhanced the migration of EPCs (Figure S8A, B). Regarding tube formation ability, EPCs were stained with Calcein‐AM, and the number of junctions and total tube lengths exhibited parallel trends (Figure 5I,J). Both cell migration and tube formation were impaired by the antagonist of CGRP, BIBN4096, thus verifying the central role of CGRP.

Collectively, our results showed that the neural bioprinted scaffolds could promote EPC angiogenesis by leveraging the effector role of the DRG through secreted CGRP. Moreover, US stimulation of neural bioprinted piezoelectric scaffolds could further amplify this process, underscoring the greater capability of the engineered skeletal interoception unit to enhance angiogenesis through an established local “sensor‐effector” circuit.

### 2.6 Neural Bioprinted Piezoelectric Scaffolds Facilitate Functional “Sensor‐Effector” Circuit and Vascularization of Bone Defect In Vivo

During the in vitro experiments, we revealed the promoting effect of piezoelectric stimulation on CGRP secretion and expression and the pro‐osteogenic and pro‐angiogenic potential of the neural bioprinted piezoelectric scaffolds. Furthermore, in vivo experiments were conducted to examine the efficacy of promoting neuro‐vascularized bone regeneration by engineering a streamlined interoception unit. In the in vivo experiment, we established a femoral defect model of rats separated into Control, DRG@Control, DRG@Piezo, US‐DRG@Non‐Piezo, and US‐DRG@Piezo groups, which were filled with the corresponding scaffolds. The last two groups underwent US stimulation at the defect site.

**FIGURE 6:**
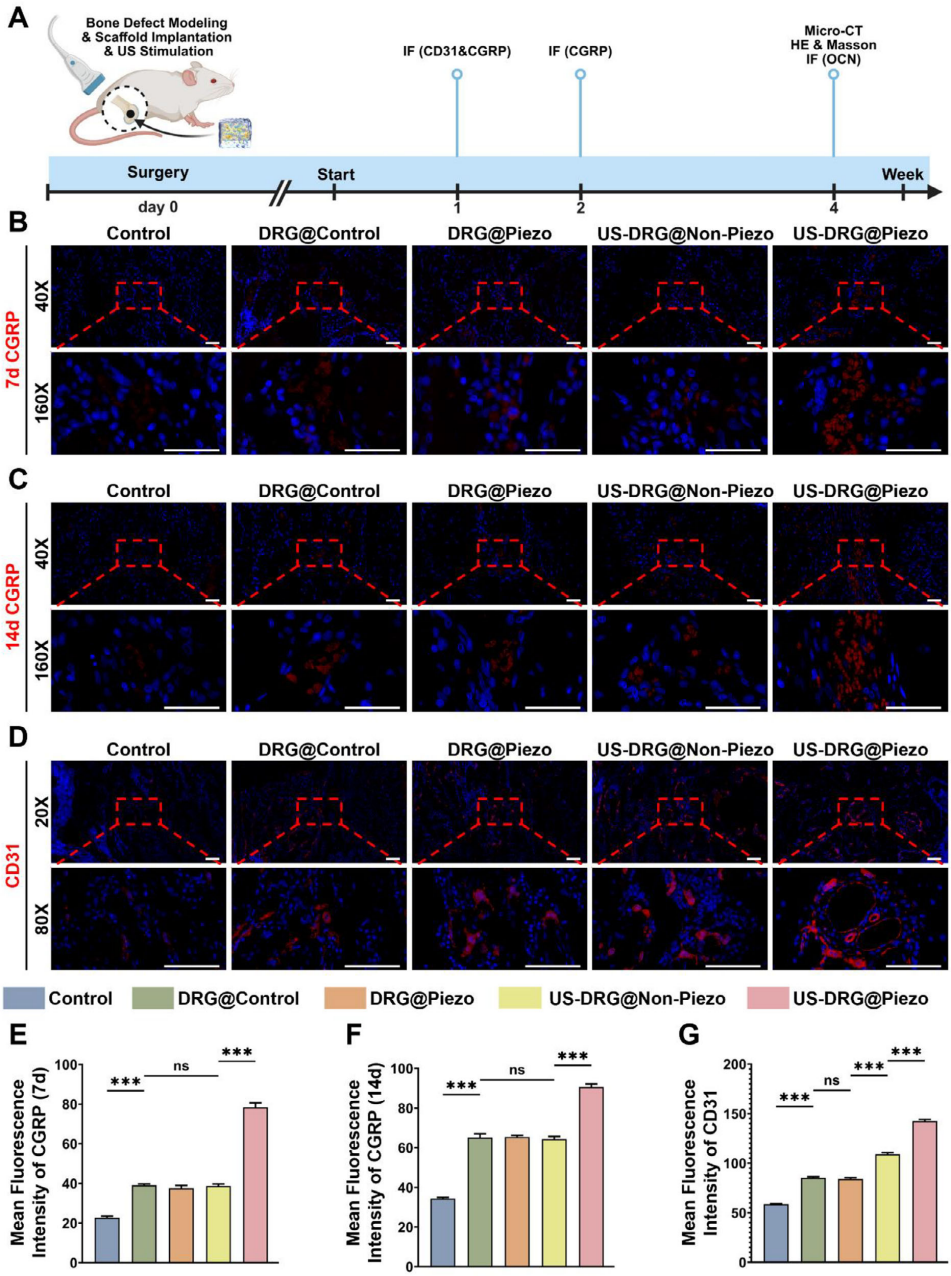
“Sensor‐effector” circuit construction and vascularization effects of the neural bioprinted piezoelectric scaffolds in bone defects in vivo. (A) Schematic illustration of in vivo bone defect modeling, scaffold implantation, US stimulation and femoral tissue harvest. (B) IF staining depicting CGRP (red) and DAPI (blue) in femoral tissue sections at 7 days from different groups: Control, DRG@ Control, DRG@ Piezo, US‐DRG@ Non‐Piezo, US‐DRG@ Piezo. (C) IF staining depicting CGRP (red) and DAPI (blue) in femoral tissue sections at 14 days from different groups. (D) IF staining depicting CD31 (red) and DAPI (blue) in femoral tissue sections at 7 days from different groups. (E) Mean fluorescence intensity of CGRP at 7 days (n = 3, mean ± s.d.). (F) Mean fluorescence intensity of CGRP at 14 days (n = 3, mean ± s.d.). (G) Mean fluorescence intensity of CD31 at 7 days (n = 3, mean ± s.d.). n = number of biologically independent samples. Scale bar, 50 µm (B, C), 100 µm (D). (ns, not significant, ^***^ p<0.0001).

To assess the capacity of DRG neurons to act as effectors incorporated in neural bioprinted piezoelectric scaffolds in vivo, femoral samples 7 and 14 days post‐surgery were collected for IF to detect the expression of CGRP (Figure 6A). As seen in Figure 6B,E, at 7days after surgery, the mean fluorescence intensity of CGRP increased significantly in the DRG@Control group compared to that in the control group. However, CGRP remained at a constant level in the DRG@Piezo group compared to the DRG@Piezo and US‐DRG@Non‐Piezo groups, ruling out the role of PLLA and US stimulation alone. In the US‐DRG@Piezo group, in which piezoelectric stimulation was applied, the CGRP expression exhibited a pronounced increase. At 14 days post‐surgery, CGRP expression remained at a relatively high level, exhibiting similar trends to those observed at 7 days post‐surgery (Figure 6C,F). To test the ability of the neural bioprinted piezoelectric scaffolds to promote vascularization, femoral samples 7 days post‐surgery were collected for IF to detect CD31 expression (Figure 6A). As seen in Figure 6D,G, at 7 days after surgery, the mean fluorescence intensity of CD31 increased significantly in the DRG@Control group compared to the control group and remained at a constant level in comparison to the DRG@Piezo group, excluding the effect of PLLA. The US‐DRG@Non‐Piezo group showed increased fluorescence intensity of CD31 compared to the DRG@Piezo group, which was possibly due to the angiogenic effect of US stimulation, whereas the US‐DRG@Piezo group demonstrated a further increasing trend under piezoelectric stimulation.

Based on the in vivo results, our study provides compelling evidence that neural bioprinted piezoelectric scaffolds enable DRG neurons to function as long‐term effector components. A key finding was the sustained release of CGRP for up to 14 days post‐implantation, which directly addresses the critical drawback of rapid CGRP degradation, which has consistently limited the efficacy of traditional CGRP‐loaded biomaterials. Notably, even in the absence of piezoelectric stimulation (DRG@Control, DRG@Piezo, and US‐DRG@Non‐Piezo groups), the neural bioprinted scaffolds demonstrated a significant increase in CGRP expression compared with the control group, indicating that the incorporated DRG neurons intrinsically possess effector functionality. The most pronounced effects are observed when neural bioprinting is combined with piezoelectric stimulation. The US‐DRG@Piezo group exhibited a substantial increase in CGRP expression, demonstrating that our strategy successfully established an engineered skeletal interoception unit capable of forming a functional local “sensor‐effector” circuit. This circuit effectively harnesses the effector capability of the incorporated DRG neurons with piezoelectric PLLA sensing and conversion of mechanical US energy into electrical signals that activate the neurons, thereby creating a responsive system inspired by natural skeletal interoception mechanisms. Moreover, the proangiogenic effects observed in vivo followed a parallel trend. While the basal effector function of DRG neurons in non‐piezoelectric scaffolds promoted vascularization to some extent, the combination with piezoelectric stimulation resulted in a significantly enhanced proangiogenic response. This synergistic effect further validated that the engineered streamlined skeletal interoception unit potently augmented the effector capability of DRG neurons through its functional “sensor‐effector” circuit, thereby promoting the vascularization of bone defects.

### 2.7 Neural Bioprinted Piezoelectric Scaffolds Promote Bone Defect Regeneration In Vivo

**FIGURE 7:**
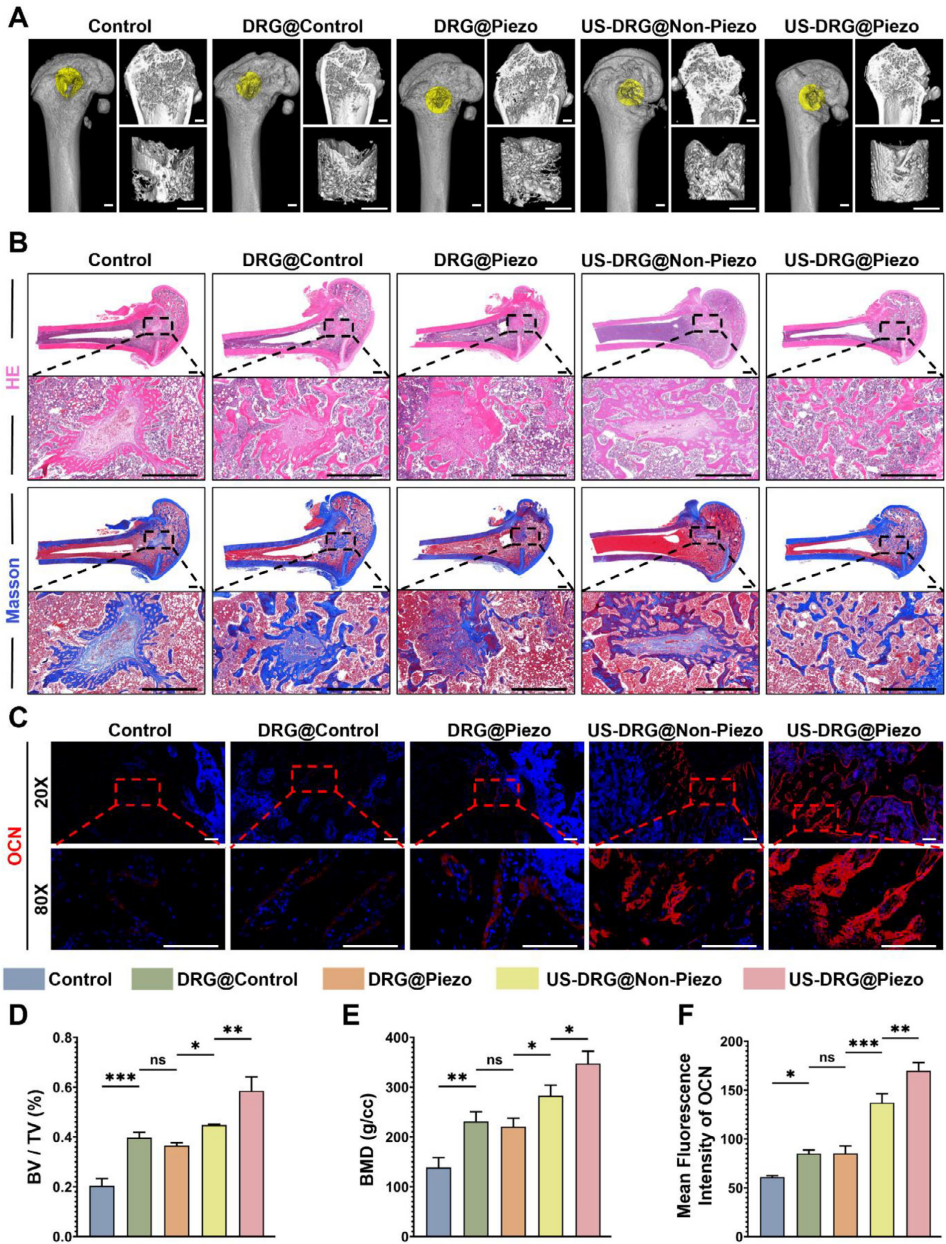
Bone regeneration effects of the neural bioprinted piezoelectric scaffolds at 4 weeks in vivo. (A) Micro‐CT images of the bone defect area in different groups: Control, DRG@Control, DRG@Piezo, US‐DRG@Non‐Piezo, US‐DRG@Piezo. (B) Full‐field view and magnified view regarding H&E and Masson staining of femoral defect in different groups. (C) IF staining depicting OCN (red) and DAPI (blue) in femoral tissue sections from different groups. (D) Quantitative analysis of BV/TV (n = 3, mean ± s.d.). (E) Quantitative analysis of BMD (n = 3, mean ± s.d.). F) Mean fluorescence intensity of OCN (n = 3, mean ± s.d.). n = number of biologically independent samples. Scale bar, 1 mm (A, B), 100 µm (C). (ns, not significant, ^*^ p<0.05, ^**^ p<0.01, ^***^ p<0.001).

To test the promotion effect of the neural bioprinted piezoelectric scaffolds on bone regeneration, femoral samples were collected 4 weeks post‐surgery for experiments including micro‐computed tomography (Micro‐CT), hematoxylin‐eosin (H&E) and Masson staining, and IF for OCN (Figure 6A). As seen in Figure 7A, which demonstrates the 3D reconstruction of Micro‐CT, a gap in the bone defect area remained evident in the control group, while it was moderately filled in the DRG@Control and DRG@Piezo groups and notably filled in the US‐DRG@Non‐Piezo and US‐DRG@Piezo groups. Quantitative analysis of bone volume fraction (BV/TV) and bone mineral density (BMD) exhibited a similar trend, with the difference in BV/TV represented by fold changes of 1.95 and 2.87, as well as an increase to 1.67‐fold and 2.50‐fold in BMD in the DRG@Control and US‐DRG@Piezo groups, respectively, when compared to the control group (Figure 7D,E). Histological assessment using H&E and Masson staining revealed decreased fibrous tissue infiltration and increased mature bone formation in the DRG@Control and DRG@Piezo groups, followed by the US‐DRG@Non‐Piezo and US‐DRG@Piezo groups (Figure 7B). The similarity between the DRG@Piezo and DRG@Control groups was probably due to PLLA's lack of osteo‐inductive capacity, whereas the difference between the US‐DRG@Non‐Piezo and DRG@Piezo groups was probably due to the osteogenic potential of US stimulation. Furthermore, the IF of OCN showed a similar trend, with a progressive increase in the fluorescence intensity from the control group to the US‐DRG@Piezo group, except for the DRG@Control and DRG@Piezo groups, which showed similar levels (Figure 7C,F).

In addition, we harvested the hearts, livers, spleens, lungs, and kidneys of rats from different groups 4 weeks after implantation of the corresponding scaffolds and analyzed them using H&E staining. The results showed no difference in the microstructure of these organs among the different groups, confirming the biocompatibility and biosafety of the neural bioprinted piezoelectric scaffolds as well as US stimulation (Figure S9).

This suggests that effector role alone of the incorporated DRG neurons in the DRG@Control group may facilitate bone regeneration. Based on this, upon piezoelectric stimulation, the US‐DRG@Piezo group, which formed a complete “sensor‐effector” circuit, presented a more pronounced bone regeneration effect. Thus, neural bioprinted piezoelectric scaffolds can promote efficient bone regeneration by constructing engineered and streamlined skeletal interoception units.

## 3 Conclusion

Guided by an innovative regenerative strategy aimed at engineering a streamlined skeletal interoception unit, we used neural bioprinting technology to fabricate neural bioprinted piezoelectric scaffolds for neuro‐vascularized bone regeneration. Key material innovations include the development of a piezoelectric bioink for neural bioprinting. The incorporated PLLA nanofibers generated on‐demand electrical stimuli under US activation as sensors, providing essential cues to promote the functionality of the DRG neurons. The incorporated DRG neurons serve as effectors that promote osteogenesis and angiogenesis via CGRP. Beyond material design, a central biological mechanism elucidated here is the Ca^2^^+^‐CGRP‐mediated coupling between neural activation and enhanced osteogenesis and angiogenesis, revealing how extrinsic piezoelectric cues promote neuro‐vascularized bone regeneration through neurosecretory pathways. Our results demonstrate that piezoelectric stimulation markedly enhances Ca^2^^+^ influx into DRG neurons, thereby facilitating synaptic vesicle‐mediated exocytosis and upregulating CGRP synthesis and release. Subsequently, the neural bioprinted piezoelectric scaffolds exhibited remarkable osteogenic and angiogenic performance, effectively driving the regeneration of the innervated and vascularized bone tissue. Overall, our work establishes a comprehensive “sensor‐effector” circuit inspired by the skeletal interoception system, forming an autonomous functional system from sensing external stimuli to exerting effector roles including enhanced secretion as well as expression of CGRP and furtherly osteotenesis as well as angiogenesis. This study provides a comprehensive and promising paradigm for neuro‐vascularized bone regeneration, successfully replicating the core function of the skeletal interoception system and translating mechanical perception into intrinsic repair responses within the engineered bone tissue.

This study has some limitations. The healthy animal model as well as primary neuronal cells used in this study may not fully replicate the commonly compromised healing environments, and face challenges including cell sources. Moreover, further investigation regarding the long‐term stability and potential immune response of the scaffold is required. Addressing these limitations in future work will facilitate the translation of this bioinspired platform toward clinical application.

## 4 Experimental Section

### 4.1 Materials

PLLA, epithelial growth medium, and poly‐d‐lysine hydrobromide were purchased from Sigma. 𝛼‐Minimum essential medium (α‐MEM), fetal bovine serum (FBS), Neurobasal medium, B27 supplement, and laminin were purchased from Gibco. GelMA and lithium phenyl (2,4,6‐trimethylbenzoyl) phosphinate (LAP) were purchased from EFL. FM1‐43 and BIBN4096 were purchased from MCE. Live/dead cell staining kit, Fluo‐4 AM, BCIP/NBT solution, and Alkaline Phosphatase Assay Kit were purchased from Beyotime. CGRP ELISA Kit was purchased from Elabscience. Osteogenic medium was purchased from Oricell. BCA Protein Assay Kit was purchased from Thermo. ARS staining kit were purchased from Amizona. Matrigel was purchased from Corning. Primary antibodies for OCN, CD31 and vWF, DAPI, and secondary antibodies were purchased from Servicebio (GB125684, GB120005, GB11020). Primary antibody for OPN was purchased from Proteintech (22952‐1‐AP). Primary antibody for CGRP was purchased from Cell Signaling Technology (14959).

### 4.2 Preparation of Aligned PLLA Fiber Membranes

A solution of 0.8 g PLLA in 10 mL hexafluoroisopropanol (HFIP) was stirred for 24 h to obtain an 8% w/v homogeneous spinning solution. The solution was loaded into a 10 mL syringe equipped with a G21 needle. Electrospinning was performed under the following parameters: voltage of 14 kV, flow rate of 1.7 mL h^−1^, needle‐to‐collector distance of 15–16 cm, rotational speed of the aluminum foil collector of 3000 rpm, and ambient humidity maintained between 25%–45%. The resulting aligned PLLA fiber membranes were air‐dried overnight to evaporate residual solvent and then stored in a vacuum desiccator for further use.

### 4.3 Preparation of PLLA Nanofibers

Aligned PLLA fiber membranes were carefully peeled from the aluminum foil and cut into approximately 0.5 cm^2^ pieces. These fragments were dispersed in 100 mL of 0.2% PVA solution and homogenized at 10 000 rpm for 3 min per cycle, repeated three times. The mixture was then filtered through a nylon membrane using a Buchner funnel to collect the preliminary fiber product. This intermediate product was redispersed in 100 mL of tert‐butanol and homogenized again at 12 000 rpm for 10 min per cycle, also repeated three times. After filtration, the resulting PLLA nanofibers were rinsed sequentially with water and ethanol, filtered again, and air‐dried overnight. The dried fibers were annealed in a muffle furnace first at 105 °C for 10 h, followed by a second annealing at 160 °C under the same duration, with gradual cooling to room temperature between steps. The final product was stored in a vacuum desiccator.

### 4.4 SEM

The microscopic morphology of the nanofibers was observed using a Phenom ProX SEM machine from the Netherlands. An appropriate amount of nanofibers was dispersed in anhydrous ethanol and thoroughly mixed with a vortex mixer. A droplet of the suspension was then deposited onto an aluminum foil and allowed to dry at room temperature. Subsequently, both the nanofibers and the fiber membranes were sputter‐coated with a layer of gold. After coating, the samples were imaged under the SEM for morphological characterization.

### 4.5 XRD

The crystal structure of the samples was analyzed using a Rigaku SmartLab SE XRD machine from Japan. The measurements were carried out under the following conditions: a scanning range of 10° to 80°, a scanning rate of 5° min^−1^, and a Cu Kα radiation source (λ = 1.5406 Å). Prior to the test, the sample was dried and evenly spread on the sample holder for measurement.

### 4.6 DSC

The thermal properties of the material were analyzed using a TA DISCOVERY DSC2500 DSC instrument. Approximately 5 mg of the sample was weighed and hermetically sealed in an aluminum crucible, with an empty crucible used as the reference. The measurement was conducted under a nitrogen atmosphere with a heating rate of 10 °C min^−1^ from 50 °C to 210 °C, and the heat flow curve was recorded.

### 4.7 Piezoelectric Coefficient Measurement

The piezoelectric properties of the samples were measured using a ZJ‐3A‐type quasi‐static piezoelectric coefficient measuring system. The samples were cut into specified dimensions and subjected to a poling treatment at room temperature under a DC voltage of 2000 V for 1 h. The d_33_ coefficient was measured immediately after the poling process.

### 4.8 Preparation of the Piezoelectric Scaffolds for Piezoelectric Output Testing

A GelMA prepolymer solution containing 0.3 wt% photoinitiator was thoroughly mixed with 0–10 mg∙mL^−^^1^ piezoelectric PLLA nanofibers at room temperature and degassed. The mixture was then injected into a polytetrafluoroethylene (PTFE) mold (custom‐sized, e.g., 1 cm × 1 cm). A flexible copper foil electrode was pre‐fixed at the bottom of the mold. Cross‐linking was achieved under 365 nm ultraviolet light at an intensity of 10 mW cm^−^^2^ for 2 min. Subsequently, another copper foil electrode of the same specification was placed on top of the scaffolds, which were subsequently insulated along the edges using Kapton tape, with only the electrode lead areas exposed for electrical connection.

### 4.9 Piezoelectric Output Testing

The encapsulated scaffold was fixed horizontally, with copper wires extending from both electrodes and connected to a Keithley 2182A nanovoltmeter (resolution: 1 nV). A 1 MHz US therapeutic instrument (probe diameter: 30 mm, calibrated power: 1 W cm^−^^2^) was applied vertically to irradiate the center of the device. US coupling gel was applied between the probe tip and the sample surface, maintaining a distance of 1 mm. Under continuous US stimulation, the open‐circuit voltage was synchronously recorded by the instrument.

### 4.10 Cell Isolation and Culture

BMSCs were harvested from bilateral femurs/tibiae of four‐week‐old male Sprague Dawley (SD) rats according to previous studies [47, 48]. After euthanasia and surface sterilization (70% ethanol), hind limbs were dissected. Bones were rinsed in ice‐cold phosphate buffer saline (PBS), ends cut, and marrow flushed into α‐MEM using a syringe. The suspension was filtered with 70 µm mesh to remove debris, and then cultured in α‐MEM + 10% FBS + 1% double antibiotics at 37°C/5% CO_2_, with medium changed every 3 days. P3–P5 cells were used for experiments. EPCs were also isolated from the bone marrow of bilateral femurs/tibiae in four‐week‐old SD rats [49, 50], using the same flushing procedure described for BMSCs isolation. Following a 48‐h incubation, non‐adherent cells were collected and transferred to a new culture dish coated with rat‐tail collagen. Primary EPCs were then cultured in endothelial growth medium, with the medium replaced every 3 days. Cells from passages P3–P5 were used for subsequent experiments. Primary sensory neurons were isolated from the DRGs of 4‐week‐old male rats through collagenase A (1 mg mL^−1^) and trypsin (0.125%) digestion, as described in previous protocols [18, 26]. The isolated cells were then resuspended and cultured in Neurobasal medium supplemented with 1% B27 supplement and plated on 6‐well plates or glass bottom cell‐culture dishes coated with poly‐d‐lysine hydrobromide (50 ng mL^−1^) and laminin (20 ng mL^−1^) at the density of 1 × 10^5^/well or 1 × 10^4^/dish respectively. Medium was changed every 3 days. DRG neurons following in vitro culturing for 6 days were used for the following experiments.

### 4.11 Bioink Preparation

GelMA was dissolved in PBS to 7% w/v. After adding 0.3 wt% LAP as photo‐initiator, the solution was incubated at 60°C until complete dissolution, filter‐sterilized (0.22 µm), and mixed with PLLA or PDLLA nanofibers (5 mg mL^−1^) to form the pre‐scaffold. This solution was pipette‐mixed, centrifuged (300 × g, 5 min) to remove bubbles, and incubated at 37°C. For bioprinting, DRG neurons (1 × 10^5^ cells/mL) were mixed into the pre‐scaffold, pipetted to homogeneity, and loaded into the bioprinting cartridge.

### 4.12 Bioprinting and Biocompatibility Assays

Bioprinting was performed using a triaxial bioprinter. The instrument was set up in an aseptic environment, surface‐sanitized with 75% ethanol and ultraviolet (UV) light, and all components (cartridges, needles, tweezers) were autoclaved (121°C, 2 h). A 15 mm × 15 mm × 2 mm grid model designed in SOLIDWORKS served as the blueprint. Printing parameters were: wire diameter = 300 µm, spacing = 300 µm, pressure = 0.2 MPa, nozzle temperature = 18°C, platform temperature = 20°C. Post‐printing, constructs were UV‐photocrosslinked (2 min), transferred to a 6‐well plate, and cultured Neurobasal supplemented with 1% B27 supplement at 37°C/5% CO_2_. Bioprinted construct biocompatibility was assessed via live/dead cell staining and CGRP ELISA. After 7 days of culture, live/dead staining was performed using the live/dead cell staining kit according to manufacturer's instructions. Samples were imaged on a laser scanning confocal microscope. Calcein AM‐stained viable cells (green fluorescence) were excited at 488 nm, while propidium iodide (PI)‐labeled dead cells (red fluorescence) were excited at 555 nm. For CGRP ELISA, medium was changed daily. After 3, 7, 14, 21 and 28 days of culture, supernatant of the scaffolds was collected for CGRP concentration testing using a CGRP ELISA Kit according to the manufacturer's instructions.

### 4.13 In Vitro Assays for Piezoelectric Stimulation on DRG Neurons

For CGRP secretion assays, DRG neurons cultured beneath scaffolds or piezoelectric neural bioprinted scaffolds were treated with non‐ or US stimulation (1 W cm^−2^, 20 min). For in vitro experiments involving US stimulation, the probe was vertically aligned and secured beneath the culture plates or dishes using a stabilized holder. Acoustic coupling gel was used at the probe‐sample interface to ensure efficient energy transfer for uniform exposure. The supernatant of the piezoelectric neural bioprinted scaffolds was then collected as the condition medium for subsequent experiments, and concentration of CGRP is detected by an ELISA Kit according to the manufacturer's instructions. CGRP expression assays were detected with qPCR or IF staining after DRG neurons were cultured beneath scaffolds with or without US stimulation (1 W cm^−2^, 20 min) for 3 consecutive days.

### 4.14 Detection of Synaptic Vesicle Exocytosis

DRG neurons were incubated with FM1‐43 fluorescence dye at room temperature for 10 min to ensure binding of the dye to cell membranes and inner membrane organelles. After incubation, scaffolds were put on top of the cells following non‐US or US stimulation (1 W cm^−2^, 20 min). Every 5 min, confocal microscopic images were acquired. False color images were generated using ImageJ.

### 4.15 Membrane Potential Measurements

The effect of piezoelectric stimulation on the membrane potential of DRG neurons was assessed using the whole‐cell patch‐clamp technique in current‐clamp mode. The cultured DRG neurons were perfused with standard extracellular solution at room temperature. The patch pipettes were pulled from borosilicate glass and filled with the intracellular solution. Pipette resistance typically ranged between 3‐5 MΩ when filled. After establishing a high‐resistance seal (>1 GΩ) and obtaining the whole‐cell configuration, the amplifier was switched to current‐clamp (I = 0) mode to monitor the membrane potential without injecting current. Changes in membrane potential in response to stimulation were recorded in real‐time, digitized, and analyzed offline.

### 4.16 Detection of Intracellular Calcium

DRG neurons were incubated with Hanks’ balanced solution supplemented with 4 × 10^−6^ M Fluo‐4 AM and 1 × 10^−6^ M Pluronic F‐127 for 30 min. Following scaffolds placing on top of the cells with non‐ or US stimulation for 20 min, DRG neurons with or without piezoelectric stimulation were observed and photographed using confocal laser microscope. The fluorescent intensity of calcium was quantified by Image J software. For calcium channels blocking, 10 µM of verapamil was added into the medium 24 h before US stimulation.

### 4.17 In Vitro Assays for Osteogenesis and Angiogenesis

To assess the effects of condition medium from piezoelectric neural bioprinted scaffolds on osteogenesis and angiogenesis, BMSCs or EPCs were cocultured with 90% osteogenic medium or 90% endothelial growth medium, respectively and 10% condition medium. For antagonism of CGRP, 10 µM of BIBN4096 were added in the medium. For CCK‐8 assays, BMSCs or EPCs were seeded in 96‐well plates at a density of 3 × 10^3^ cells/well and cocultured for 1, 3 and 5 days. For qPCR assays, BMSCs or EPCs were seeded in 6‐well plates at a density of 2 × 10^5^ cells/well and cocultured for 3 days. For IF staining assays, BMSCs or EPCs were seeded in glass bottom cell‐culture dishes at a density of 1 × 10^4^ cells/well and cocultured for 7 or 3 days, respectively.

### 4.18 ALP Staining, ALP Activity Testing and ARS Staining

For ALP staining, BMSCs were seeded in 24‐well plates at a density of 5 × 10^4^ cells/well. After coculture for 14 days, BMSCs were fixed for 15 min in 4% paraformaldehyde and stained with BCIP/NBT solution at room temperature for 30 min. After rinsing three times with PBS, the cells were observed and photographed under a microscope. For ALP activity testing, BMSCs were seeded in 6‐well plates at a density of 2 × 10^5^ cells/well. After coculture for 14 days, BMSCs were harvested followed by analysis through Alkaline Phosphatase Assay Kit and BCA Protein Assay Kit following the manufacturer's standard procedure, and absorbance was measured at 405 and 562 nm using a microplate reader. For evaluation of the formed mineralized matrix nodules, BMSCs were seeded in 24‐well plates at a density of 5 × 10^4^ cells/well. After coculture for 21 days, BMSCs were fixed for 15 min in 4% paraformaldehyde and stained with Alizarin Red S solution at room temperature for 30 min. After rinsing three times with PBS, the cells were observed and photographed under a microscope. To quantify mineralization, mineralized bone nodules were stained with 10% cetylpyridinium chloride and measured at 562 nm using a microplate reader.

### 4.19 Transwell Migration Assays

EPCs were seeded in the upper chambers of Transwell inserts at the density of 2 × 10^5^ cells/insert, with the lower chambers filled with 90% serum‐free endothelial growth medium and 10% corresponding condition medium or control. After 24 h, the transwell inserts were removed, fixed for 30 min in 4% paraformaldehyde, with the cells remained on upper side of the membranes removed with a cotton swab, and stained with 0.1% crystal violet solution at room temperature for 30 min. The stained cells were observed and photographed using a microscope, followed by analysis by image J software.

### 4.20 Tube Formation Assays

EPCs were seeded in glass bottom cell‐culture dishes pretreated with Matrigel at a density of 2.5 × 10^5^ cells/dish. After coculture for 6 h, tubes formed by EPCs were observed and photographed using a microscope. Analysis of number of junctions and total tube lengths was performed using image J software.

### 4.21 CCK‐8 Assays

Upon reaching the respective endpoints, the culture medium in each well was replaced with fresh medium containing 10% CCK‐8 reagent. Following incubation at 37°C for 1 h, the absorbance was measured at 450 nm with a microplate reader.

### 4.22 qPCR Assays

Total RNA of DRG neurons, BMSCs and EPCs was extracted by RNA Purification Kit, and then reversely transcribed into cDNA by the First Strand cDNA Synthesis Kit. After that, Real‐time quantitative PCR reaction using 2^−ΔΔCt^ method was performed with cDNA as template using Taq Pro Universal SYBR qPCR Master Mix. Primer sequences used are listed in Table S1.

### 4.23 IF Staining Assays

DRG neurons, BMSCs and EPCs were fixed for 15 min in 4% paraformaldehyde and then incubated overnight with βIII‐Tubulin as well as CGRP antibodies, OCN or OPN antibodies, and CD31 or vWF antibodies, followed by secondary antibodies and DAPI. The fluorescent intensity was quantified by Image J software.

### 4.24 In Vivo Surgical Procedures

All animal experiments in this study were approved by the Animal Research Committee of Xinhua Hospital, Shanghai Jiao Tong University School of Medicine (Approval ID: XHEC‐F‐2025‐049). All animals were purchased from Shanghai ShengChang Biotechnology Limited Liability Company. Following anesthesia with 3% pentobarbital sodium, we created a rat model featuring 3‐mm femoral condyle defects using a previously reported method to evaluate bone regeneration [51, 52]. 45 male 8‐week old SD Rats (weighing 290–310 g) were separated into 5 groups (n = 9) including: (1) Control group: implantation of the Control scaffold at the defect site; (2) DRG@Control group: implantation of the DRG@Control scaffold; (3) DRG@Piezo group: implantation of the DRG@Piezo scaffold; (4) US‐DRG@Non‐Piezo group: implantation of the DRG@Non‐Piezo scaffold along with US stimulation at the defect site; (5) US‐DRG@Piezo group: implantation of the DRG@Piezo scaffold along with US stimulation at the defect site; For the last two groups, US stimulation (1 W cm^−2^, 20 min) was applied three times a week. For in vivo US stimulation, after shaving and cleaning the area, the probe was placed perpendicular to the skin surface overlying the femoral condyle defect. A medical‐grade ultrasound coupling gel was applied, and the probe was held in a fixed position using a mechanical holder to ensure consistent exposure across all animals. At the experimental endpoint, rats were sacrificed, with femoral bone tissues as well as major organs including hearts, livers, spleens, lungs and kidneys harvested and fixed in 4% paraformaldehyde for subsequent analysis.

### 4.25 Micro‐CT

New bone formation of femoral samples was quantified using Micro‐CT imaging system. A cylindrical region of interest (5 mm diameter × 2 mm depth) was defined for bone regeneration analysis prior to 3D reconstruction. BV/TV and BMD were quantified using Scanco software.

### 4.26 IF Staining and Histological Analysis

Following Micro‐CT scanning, femoral samples underwent decalcification, graded ethanol dehydration, and paraffin embedding. Sections (≈5 µm) were prepared for subsequent staining. For IF staining, femoral sections were incubated with CGRP antibody, CD31 antibody, or OCN antibody overnight at 4°C, followed by secondary antibodies and DAPI. The mean fluorescence intensity of CGRP, CD31 and OCN was quantified by Image J software. For histological analysis, femoral and organ sections underwent H&E and Masson staining.

### 4.27 Statistical Analysis

All experiments were performed in triplicate. Data are presented as Mean ± SD. Comparisons between two groups used unpaired two‐tailed t‐tests. Multiple comparisons were analyzed by one‐way ANOVA with Tukey's post‐hoc test. Analyses were conducted in GraphPad Prism 9. Significance thresholds: * p < 0.05, ** p < 0.01, * p < 0.001; ns (not significant) = p > 0.05.

## Funding

(This work was supported by National Key Research and Development Program of China (2023YFC2411304), National Natural Science Foundation of China (82172473, 32401133, 82272486, and 82572771), Shanghai Municipal Commission of Science and Technology (24YF2701400), Donghua University 2025 Cultivation Project of Discipline Innovation (xkcx‐202512)), Natural Science Foundation of Hunan Province (2023JJ30749), and China Postdoctoral Science Foundation (2023M7427373 and 2025M781698).

## Ethics Approval

All Animal Experiments in This Study Were Approved By the Animal Research Committee of Xinhua Hospital, Shanghai Jiao Tong University School of Medicine (Approval ID: XHEC‐F‐2025‐049).

## Conflicts of Interest

The authors declare no conflicts of interest.

## Supporting information




**Supporting File**: advs74238‐sup‐0001‐SuppMat.docx

## Data Availability

The data that support the findings of this study are available from the corresponding author upon reasonable request.
