# Supplementary material for: Bioinspired Engineering of Streamlined Skeletal Interoception: Neural Bioprinted Piezoelectric Scaffolds for Neuro‐Vascularized Bone Regeneration
Source: Adv Sci (Weinh). 2026 Feb 15;13(21):e24181. doi: 10.1002/advs.202524181 (PMC13073224; doi:10.1002/advs.202524181)
Supplement: Supplementary file 1 — Supporting File: advs74238‐sup‐0001‐SuppMat.docx [file ADVS-13-e24181-s001.docx]

Supporting Information

Bioinspired Engineering of Streamlined Skeletal Interoception: Neural Bioprinted Piezoelectric Scaffolds for Neuro-Vascularized Bone Regeneration

*Yingze Su, Haomin Wang, Weixi Liu, Kangming Chen, Yiyang Min, Jinbo Zhu, Xueyang Li, Anning Su, Hao Yang, Lei Yang, Yun Ji, Yuxin Zhang, Jisi Zheng, Chi Yang, Chuanglong He, Tao Li*, Shuo Chen*, Tao Wu*, Xiaodong Chen**

Table S1 Primers sequences used for qPCR

| **Gene** | **Forward primers (5’ to 3’)** | **Reverse primers (5’ to 3’)** |
| --- | --- | --- |
| CGRP | CAGTGAAGAAGAAGCTCGCCTAC | CTCAGCCTCCTGTTCCTCCTC |
| RUNX2 | TCGTCAGCGTCCTATCAGTTCC | CTTCCATCAGCGTCAACACCATC |
| ALP | ACTGATGTGGAATATGAACTGGATGAG | ATAGTGGGAGTGCTTGTGTCTAGG |
| OCN | GACCCTCTCTCTGCTCACTCTG | CACCACCTTACTGCCCTCCTG |
| OPN | CGATGATGACGACGACGATGAC | CTTGTGTGCTGGCAGTGAAGG |
| CD31 | GCCAGCATTGTGACCAGTCTC | CAAGGCGGCAATGACCACTC |
| Hif-1α | GTGAGCACAGTTACAGGATTCCAG | TGGCGGTGGTGGCAGTTG |
| vWF | GGACGCACATCGCAGTTCTG | TCGCAGCACATCCTCCTTGG |
| VEGF | TGACGGACAGACAGACAGACAC | GGGCTTCGGCTCCTGCTC |
| GAPDH | AAGTTCAACGGCACAGTCAAGG | GACATACTCAGCACCAGCATCAC |


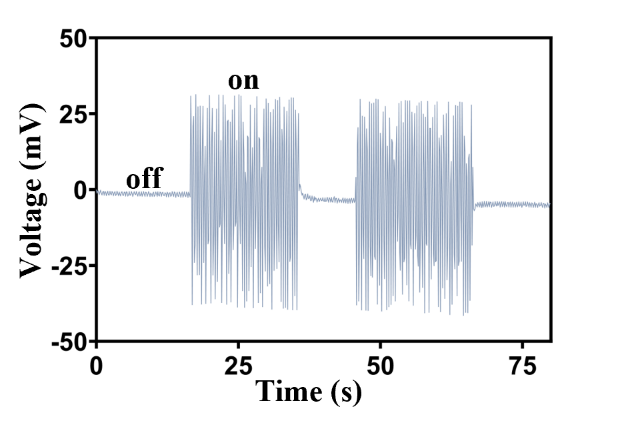


**Figure S1.** US-responsive voltage output testing of the piezoelectric scaffolds.


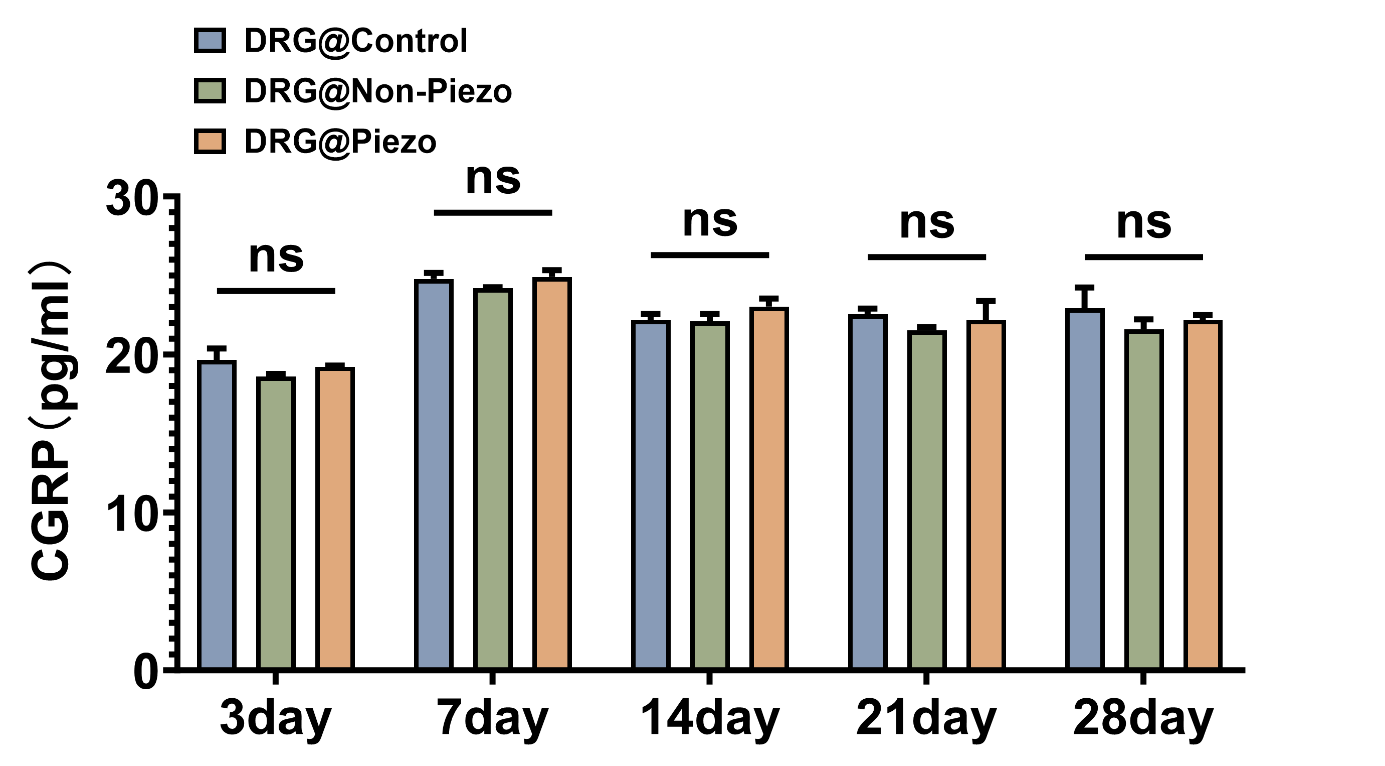


**Figure S2.** ELISA for CGRP concentration of DRG@Control, DRG@Non-Piezo and DRG@Piezo scaffolds after culturing for 3, 7, 14, 21 and 28 days (n = 3, mean ± s.d.). n = number of biologically independent samples. (ns, not significant).


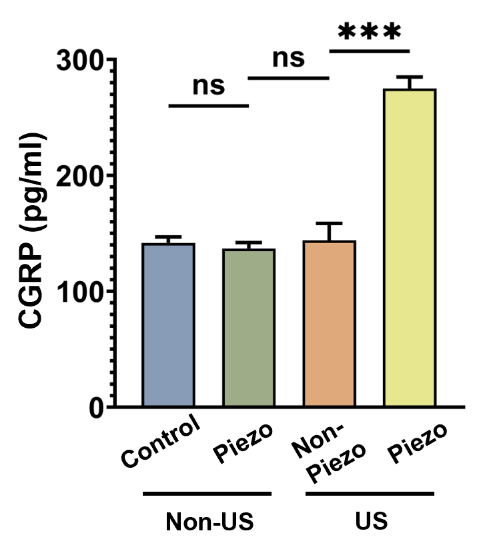


**Figure S3.** ELISA for CGRP concentration of DRG neurons cultured beneath Control, Piezo and Non-Piezo scaffolds with or without US stimulation (n = 3, mean ± s.d.). n = number of biologically independent samples. (ns, not significant, *** p < 0.001).


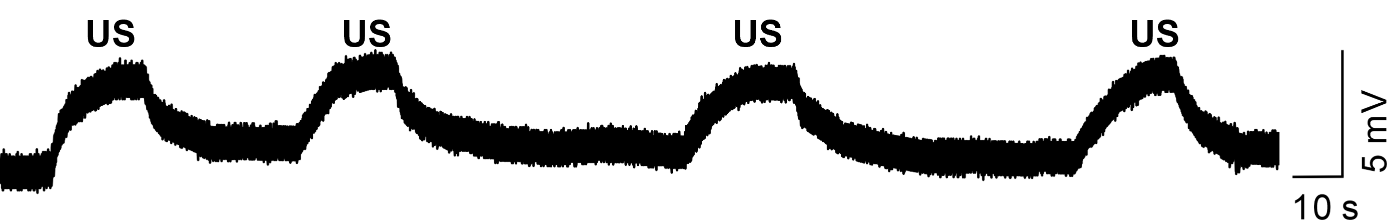


**Figure S4.** Membrane potential traces of DRG neurons with or without US-induced piezoelectric stimulation.


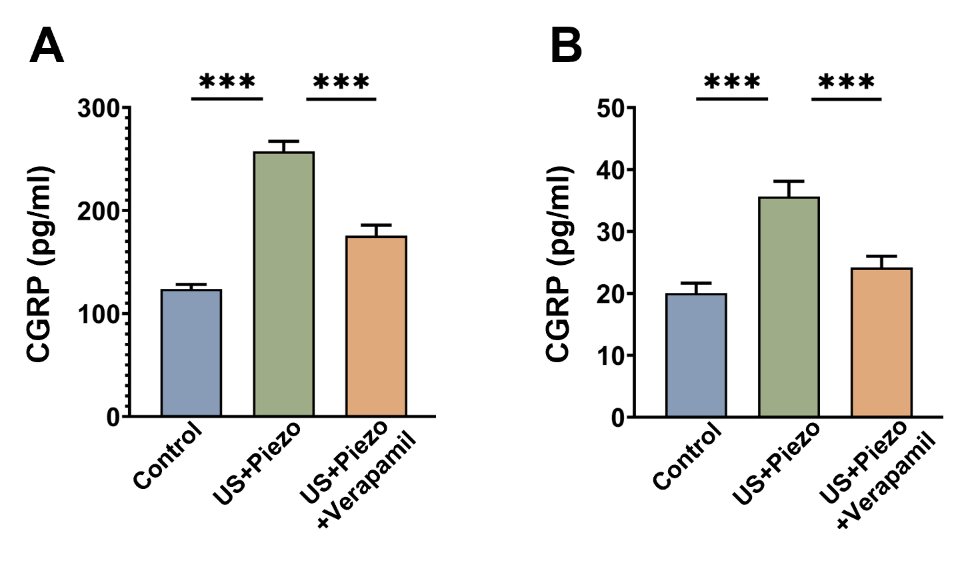


**Figure S5.** ELISA for CGRP concentration of different groups: Control, US+Piezo, and US+Piezo+Verapamil. A) ELISA for CGRP concentration of DRG neurons cultured beneath scaffolds from different groups (n = 3, mean ± s.d.). B) ELISA for CGRP concentration of neural bioprinted scaffolds from different groups (n = 3, mean ± s.d.). n = number of biologically independent samples. (*** p < 0.001).


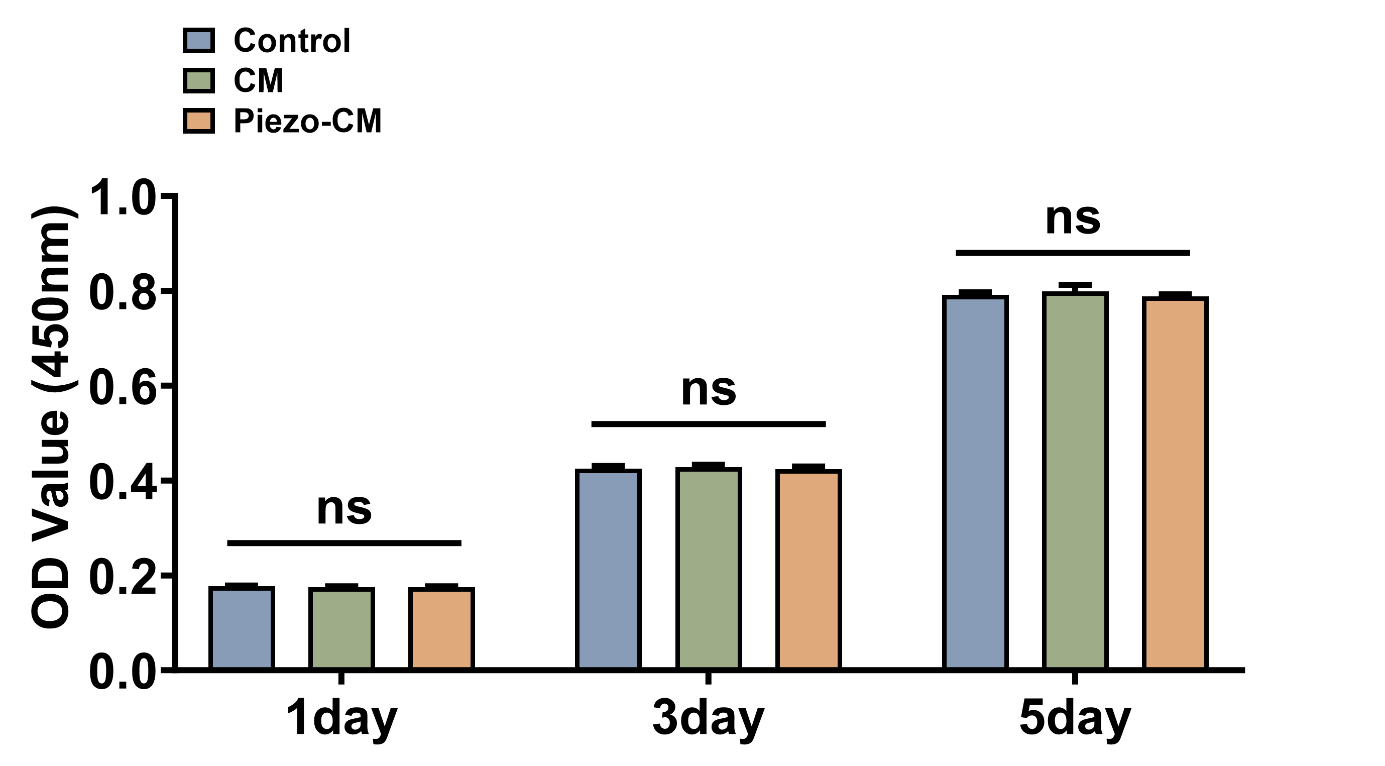


**Figure S6.** CCK-8 assay for viability of BMSCs at 1, 3 and 5 days from different groups: Control, CM, and Piezo-CM (n = 3, mean ± s.d.). n = number of biologically independent samples. (ns, not significant).


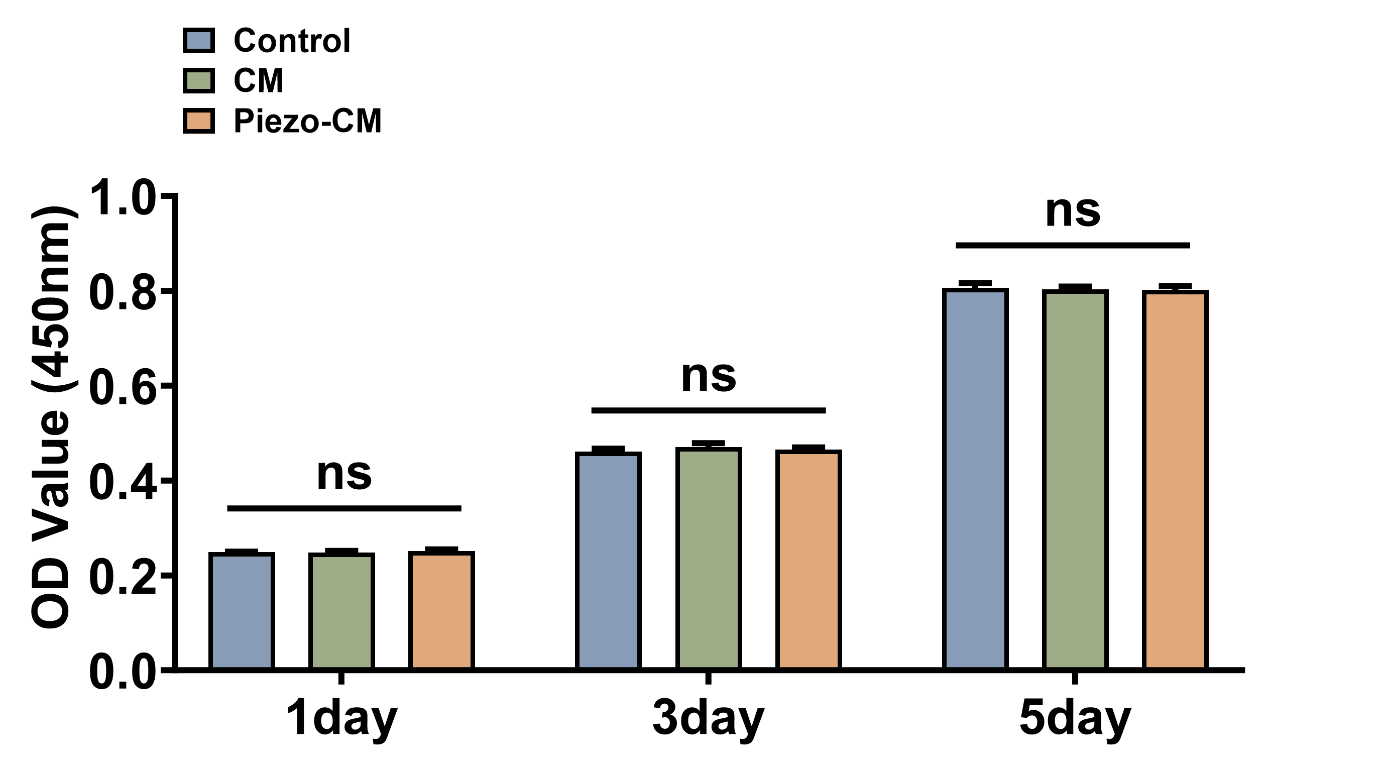


**Figure S7.** CCK-8 assay for viability of EPCs at 1, 3 and 5 days from different groups: Control, CM, and Piezo-CM (n = 3, mean ± s.d.). n = number of biologically independent samples. (ns, not significant).


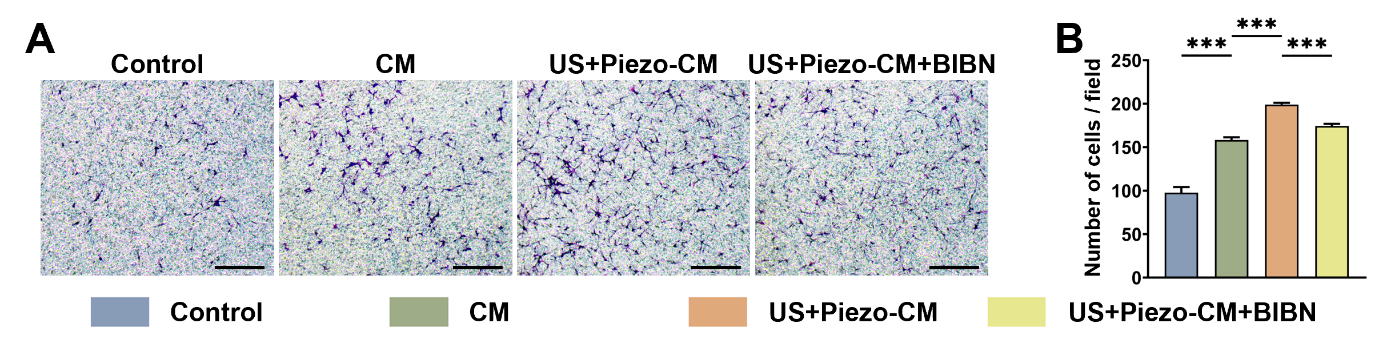


**Figure S8.** Transwell migration assay of EPCs from different groups: Control, CM, US+Piezo-CM, US+Piezo-CM+BIBN4096. A) Migrated EPCs stained with crystal violet solution. B) Analysis of migrated cells per field (n = 3, mean ± s.d.). n = number of biologically independent samples. Scale bar, 400 μm (B). (*** p < 0.001).


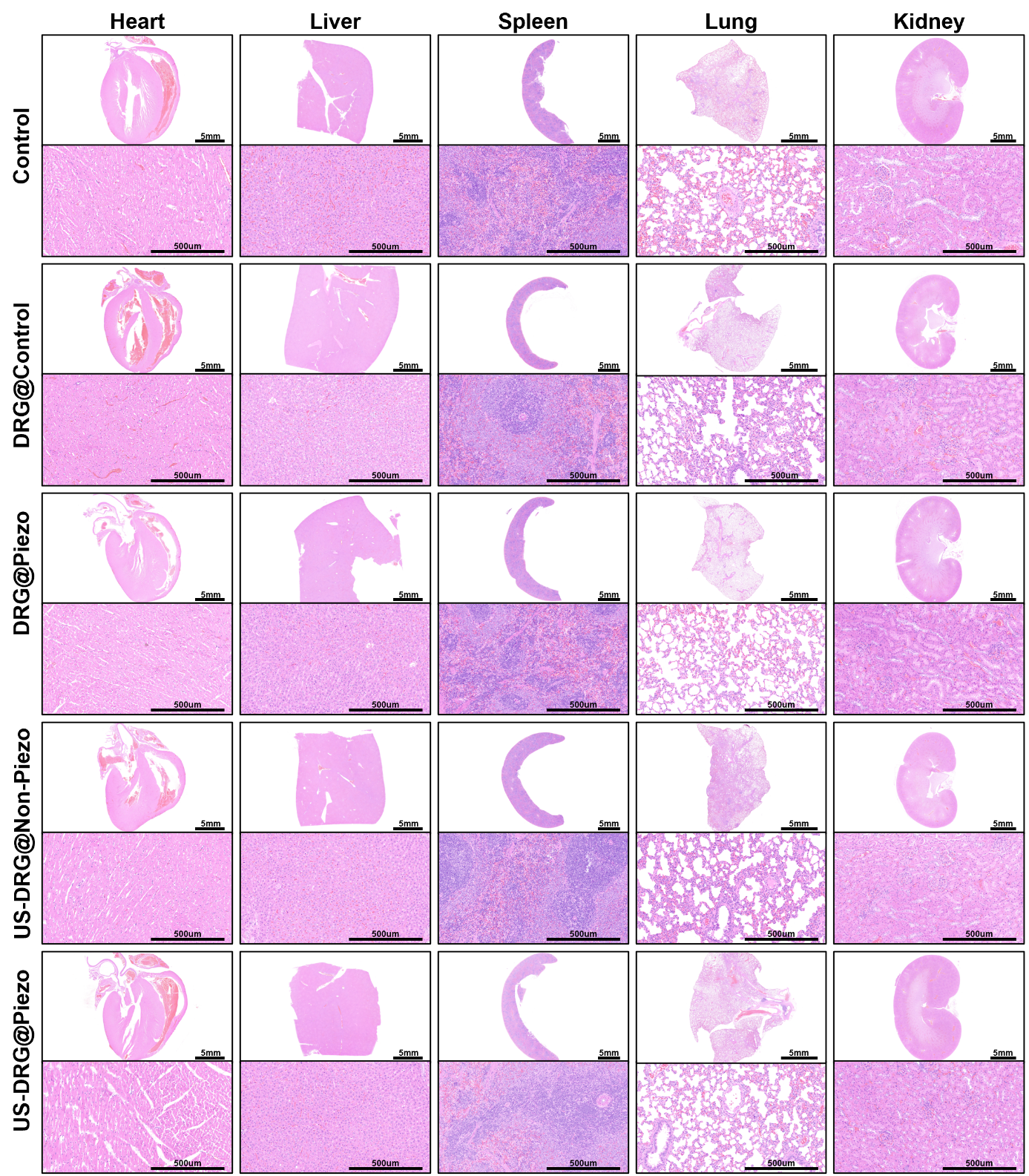


**Figure S9.** In vivo biocompatibility and biosafety of the neural bioprinted piezoelectric scaffolds. H&E staining was performed on histological sections from major organs, including the hearts, livers, spleens, lungs and kidneys.
